# Whole body synthesis rates of DHA from α-linolenic acid are greater than brain DHA accretion and uptake rates in adult rats[S]

**DOI:** 10.1194/jlr.M042275

**Published:** 2014-01

**Authors:** Anthony F. Domenichiello, Chuck T. Chen, Marc-Olivier Trepanier, P. Mark Stavro, Richard P. Bazinet

**Affiliations:** * Department of Nutritional Sciences, University of Toronto, Toronto, Ontario M5S 3E2, Canada; † Bunge Ltd., White Plains, NY 10606

**Keywords:** brain, docosahexaenoic acid, kinetics, a-linolenic-acid, liver, synthesis, conversion

## Abstract

Docosahexaenoic acid (DHA) is important for brain function, however, the exact amount required for the brain is not agreed upon. While it is believed that the synthesis rate of DHA from α-linolenic acid (ALA) is low, how this synthesis rate compares with the amount of DHA required to maintain brain DHA levels is unknown. The objective of this work was to assess whether DHA synthesis from ALA is sufficient for the brain. To test this, rats consumed a diet low in n-3 PUFAs, or a diet containing ALA or DHA for 15 weeks. Over the 15 weeks, whole body and brain DHA accretion was measured, while at the end of the study, whole body DHA synthesis rates, brain gene expression, and DHA uptake rates were measured. Despite large differences in body DHA accretion, there was no difference in brain DHA accretion between rats fed ALA and DHA. In rats fed ALA, DHA synthesis and accretion was 100-fold higher than brain DHA accretion of rats fed DHA. Also, ALA-fed rats synthesized approximately 3-fold more DHA than the DHA uptake rate into the brain. This work indicates that DHA synthesis from ALA may be sufficient to supply the brain.

α-Linolenic acid (ALA) is the most accessible and sustainable source of omega-3 polyunsaturated fatty acids (n-3 PUFAs) in the global diet (1). ALA is also a precursor to docosahexaenoic acid (DHA), an n-3 PUFA that is particularly enriched within the brain (2). While it is generally accepted that DHA is important for normal brain function, the amount of DHA required by the brain is not agreed upon (3–7). n-3 PUFAs cannot be synthesized by mammals de novo, therefore, DHA must be consumed from dietary sources or be synthesized from shorter chain n-3 PUFAs (i.e., ALA). To date, reports suggest that the synthesis rate of DHA from ALA is low and perhaps even below detection (8–17). However, plasma concentrations of DHA in vegans are only 0–40% lower than fish eaters despite having no dietary DHA (18, 19). Furthermore, vegan and vegetarian populations do not have an increased risk of neurological disorders (20, 21).

The lack of concordance between the low DHA synthesis rates and the relatively normal plasma DHA concentrations in vegans may be due to the methods used to measure DHA synthesis (22). In humans, an ALA tracer is administered orally and the appearance of labeled DHA in the plasma is measured for up to 2 weeks (8–17). From the area under the curve (AUC) of labeled DHA appearance, the fractional conversion of DHA is calculated (8–12, 15). Alternatively, plasma labeled DHA concentrations over time can be used in modeling programs to determine fractional conversion (13, 16, 17). In both cases, the calculations may preclude a quantitative measurement of the DHA synthesis rate and allow only a comparison between study groups (22). Another concern is the finding that up to 57% of the tracer remains in the adipose after oral consumption of labeled ALA (15). Because the human adipose half-life can be longer than one year, it is possible that the tracer is unavailable for DHA synthesis during the study period (23).

Recently, Rapoport and colleagues developed a new method in rats to estimate the DHA synthesis rate from ALA (24). This method requires a steady-state infusion of labeled ALA and uses nonlinear regression to determine the DHA synthesis rate. Importantly, this method estimates the DHA synthesis rate in rats to be 9.8 μmol/day (24), matching the 11 μmol/day synthesis and accretion rate of longer chain n-3 PUFAs, which we estimated from a published balance study performed in growing rats (25). The steady-state infusion method may have advantages because: *i*) infusing a tracer to achieve steady-state in the plasma eliminates issues around adipose tissue storage of the tracer; and *ii*) it allows for a quantitative determination of the DHA synthesis rate.

The goal of this study was to determine if DHA synthesis from ALA can maintain brain DHA in rats. We addressed this objective by measuring: *i*) brain and whole body DHA accretion; *ii*) DHA synthesis rates from ALA; and *iii*) brain DHA uptake rates in rats fed three different diets: a control diet (low n-3 PUFAs), an ALA diet (2% ALA), or a DHA diet (2% DHA). We found that rats fed ALA and DHA accreted similar amounts of brain DHA, which together with our kinetic findings suggest DHA synthesis from ALA is likely sufficient to maintain brain DHA levels. We also mimicked, in rats, the methods used in humans to determine DHA synthesis from ALA by subjecting rats to a gavage with labeled ALA. We found that the rates from this experiment, in rats, were comparable to the results of previously published human studies. Collectively, these results indicate that the rat is an appropriate model for measuring brain DHA synthesis and that brain DHA can be supplied from dietary ALA.

## MATERIALS AND METHODS

### Animals

All procedures were performed in accordance with the policies set out by the Canadian Council on Animal Care and were approved by the Animal Ethics Committee at the University of Toronto. Three Long Evans dams each with 18-day-old male Long Evans pups were ordered from Charles River Laboratories (St. Constant, QC, Canada). Pups were nonlittermates and each dam was housed with pups. Upon arrival, dams were allocated to one of the three diets (described below). When the pups were 21 days old, two pups from each dam were euthanized by high-energy head-focused microwave fixation (4.5 kW for 0.9 s). Brains were removed immediately and stored at −80°C until lipid analysis. Bodies were also stored at −80°C immediately after euthanization until analysis. The brains and bodies of these pups were used to determine the PUFA content of the rats at weaning (i.e., baseline PUFA content).

The remaining pups were weaned, singly housed, continued on the diet of their respective dam for 15 weeks, and were allocated to either the balance study (n = 11), the steady-state infusion study (n = 4), or the brain DHA uptake study (n = 3). For the balance study, the rats were weighed, food intake was determined, and uneaten food was replaced with fresh food on a weekly basis. Rats were euthanized by CO_2_ asphyxiation. Brains were removed and dissected sagittally. Carcasses and one half brain per rat were stored immediately at −80°C (to be later analyzed for lipid content). The other half brain was flash-frozen with 2-methylbutane on dry ice, and then stored at −80°C until analyzed for mRNA expression. The PUFA content in the brains and bodies of these rats after the 15 week feeding period was compared with the baseline PUFA content (described above) to determine PUFA accretion (equation 1, described below). For the steady-state infusion study and the brain DHA uptake study, a jugular vein catheter was surgically implanted into the rats. After allowing a 24 h recovery, rats were infused with ^2^H_14_-ALA or ^14^C-DHA, respectively (described below). The ^2^H_14_-ALA infusion was used to determine DHA synthesis rates following a previously published method (24) and the ^14^C-DHA infusion was utilized to measure the brain DHA uptake rate by following previously published methods (26).

For the gavage study (n = 4), a carotid artery catheter was surgically implanted in rats that had consumed a diet with ALA but no DHA for 9 weeks postweaning. After a 24 h recovery, the rats were gavaged with 10 mg of ^2^H_5_-ALA (described below).

### Diets

Diets were modified from the custom low n-3 AIN-93G purified rodent diet (Dyets Inc., Bethlehem, PA) (27). The diet contained 10% lipids (by weight). The fat content of the diet was 32.8% (by weight) safflower oil, 65.2% hydrogenated coconut oil, and 2% added oil. The added oil was either DHA ethyl ester (Equateq, Callanish, Scotland), ALA ethyl ester (Equateq), or oleate ethyl ester (Nucheck Prep, Elysian, MN). Each oil was determined to be >98% pure by gas chromatography-flame ionization detection (GC-FID) and each diet contained one added oil to make the DHA, ALA, and control diets, respectively. The custom low n-3 AIN-93G diet is designed to be deficient in n-3 PUFAs, and as a result the only n-3 PUFAs in the diets were those added as ethyl ester oils (DHA or ALA) and a residual amount of ALA that made up 0.25% of the fatty acids. Oleate ethyl ester was added to the control diet to keep total fat content of the diets consistent, and to ensure a constant n-6 PUFA level across all three diets. The fatty acid composition of each diet as measured by GC-FID as shown in **Table 1**.

**TABLE 1.** Percent composition of the three diets

Fatty Acid	Control Diet	ALA Diet	DHA Diet	Gavage Study Diet
10:0	3.6	4.0	3.8	ND
12:0	36.3	37.4	37.2	ND
14:0	15.1	14.8	14.8	0.1
16:0	10.0	9.7	9.9	14.3
16:1n-7	0.1	0.1	0.1	0.16
18:0	7.4	7.1	7.3	3.5
18:1n-9	7.5	4.6	4.7	21.8
18:1n-7	0.2	0.2	0.2	1.2
18:2n-6	19.5	19.1	20.1	52.5
18:3n-3	0.25	2.25	0.24	5.3
DHA (22:6 n-3)	ND	ND	2.00	ND

Data shown are means (n = 3) expressed as percent of total fatty acids. ND, fatty acid concentration below limit of detection.

The composition of the diet consumed by rats for the gavage study is shown in Table 1. This diet contained 52 and 5% of the fatty acids as linoleic acid (LNA) and ALA, respectively, with all other PUFAs <0.5%.

### Balance study: whole body fatty acid extraction

Carcasses were thawed overnight in a refrigerator at 3°C, cut into pieces, and passed through a #12 hand grinder. After the whole body had passed through the grinder once, the homogenate was mixed together by hand and passed at least one more time through the grinder until the mixture was sufficiently homogeneous. Due to the small size of the pups all of the 21-day-old pup bodies were passed through the grinder together.

A portion of the whole body homogenate was weighed and further homogenized (done in duplicate for each 18-week-old rat and the baseline homogenate) using a Polytron benchtop homogenizer (Brinkman Instruments, Toronto, ON, Canada) in a mixture of chloroform:methanol:0.88%KCl (2:1:0.8 by volume) (28). The mixture was centrifuged at 500 *g* for 10 min, and the chloroform layer was extracted. New chloroform was added to the remaining aqueous phase, the mixture was once again centrifuged, and the chloroform layer was extracted and added to the previously extracted chloroform layer. This total lipid extract (TLE) was evaporated under N_2_, reconstituted in a known volume of chloroform, and stored under N_2_ at −80°C until further analysis.

### Balance study: brain fatty acid extraction

One hemisphere was thawed briefly and dissected over ice to separate the brainstem, cerebellum, cortex, hippocampus, striatum, and the rest of the brain. Total lipids were extracted from the whole brain of 21-day-old rats and each region of the dissected 15 week half brains by following the method of Folch, Lees, and Sloane Stanley (28). Briefly, whole brains or brain regions were weighed in glass mortars, homogenized with glass pestles in 0.88% KCl, and transferred into a clean test tube. The homogenizer was then washed with methanol, which was then transferred to the test tube containing the homogenized brain and KCl. The homogenizer was washed a final time with chloroform and the chloroform was transferred to the test tube containing the KCl and methanol mixture (chloroform:methanol:0.88% KCl, 2:1:0.8 by volume). The homogenate mixtures were left at 3°C overnight and were centrifuged at 500 *g* for 10 min the following morning. The chloroform layer was extracted and new chloroform was added to the aqueous phase. This mixture was centrifuged at 500 *g* for 10 min and the chloroform layer was extracted and added to the previously extracted chloroform phase.

### Balance study: fecal PUFA analysis

At 8 weeks postweaning all the feces produced by a rat in 24 h were collected. Approximately 1 g of feces from 6 rats was then analyzed for PUFA content. Fatty acids were extracted with chloroform:methanol:0.88% KCl (2:1:0.8 by volume) as described above and quantified by GC-FID to determine an estimate of n-3 and n-6 PUFA fecal excretion. Mean PUFA excretion (percentage of intake) was determined and applied to all animals to determine PUFA excretion.

### Transmethylation and GC-FID

A known amount of heptadecanoic acid (17:0; Nu-Check Prep, Elysian, MN) was added to the TLE. Samples were transmethylated using 14% boron trifluoride in methanol at 100°C for 1 h. Fatty acid methyl esters (FAMEs) were extracted with hexane and quantified using GC-FID. FAMEs were analyzed using a Varian-430 gas chromatograph (Varian, Lake Forest, CA) equipped with a Varian FactorFour capillary column (VF-23ms; 30 m × 0.25 mm interior diameter × 0.25 μm film thickness) and a FID. Samples were injected in splitless mode. The injector and detector ports were set at 250°C. FAMEs were eluted using a temperature program set initially at 50°C for 2 min, increasing at 20°C/min, and held at 170°C for 1 min, then increased at 3°C/min and held at 212°C for 5 min to complete the run at 32 min. The carrier gas was helium, set to a constant flow rate of 0.7 ml/min. Peaks were identified by retention times of authentic FAME standards (Nu-Chek Prep). The concentration of each fatty acid was calculated by comparison with the internal standard (17:0) (29). The concentrations were expressed on a per gram basis then multiplied by total weight of the tissue to determine the total amount of each fatty acid in the tissue.

### Balance study: brain RNA extraction

RNA was extracted from the flash-frozen half brains of the 15 week postweaning rats. The brain was briefly thawed and dissected to isolate the brainstem, cerebellum, cortex, hippocampus, striatum, and the rest of the brain. RNA was extracted from the brain regions using Trizol reagent (Ambion; Life Technologies, Burlington, ON, Canada) according to the manufacturer's protocol. The tissues were placed in a volume of Trizol reagent that was 10 times greater than the volume of the tissue and homogenized using a Kontes tissue grinder with plastic pestles (Daigger, Vernon Hills, IL), or for larger regions, a TissueRuptor (Qiagen, Germantown, MD). Chloroform was added to the Trizol reagent at a ratio of 1:5 (chloroform:Trizol reagent) and the solution was mixed and incubated at room temperature. The solution was then centrifuged at 12,000 *g* for 15 min at 4°C. The aqueous phase was transferred into a new tube, mixed with isopropanol (1:1, isopropanol:original volume of Trizol reagent), and incubated for 10 min. The samples were then centrifuged at 12,000 *g* for 10 min at 4°C to precipitate the RNA pellet. Following the precipitation of the RNA pellet, isopropanol was removed and the pellet was washed with 75% ethanol (1:2, ethanol:original volume of Trizol reagent). The samples were then centrifuged at 7,500 *g* for 5 min at 4°C and the wash was discarded. The pellet was allowed to air dry, and was then dissolved in RNase-free water. A Nanodrop 1000 (NanoDrop Technologies Inc., Wilmington, DE) was used to determine the concentration and purity of each sample by measuring absorbance at 260 and 280 nm. Each sample was aliquoted to make 1 μg of RNA per 10 μl of sample and stored at −80°C.

### Balance study: gene expression analysis

A high-capacity cDNA reverse transcription kit (Applied Biosystems, Burlington, ON, Canada) was used to reverse transcribe 1 μg of RNA according to the manufacturer's instructions. Newly synthesized cDNA was stored at −20°C until the following day when it was loaded onto TaqMan low density array (TLDA) plates.

Quantitative real-time PCR was performed using TLDA (Applied Biosystems) on the 7900HT real-time PCR system (Applied Biosystems). cDNA (50 ng) was diluted with water to make a volume of 50 μl and mixed with 50 μl of TaqMan Universal PCR Master Mix (Applied Biosystems). The mixture was then loaded onto a customized preconfigured 384-well TLDA plate according to the manufacturer's protocol. TaqMan gene expression assays were used to assess 21 genes that were previously reported to be differentially expressed with n-3 deprivation. Among these were genes involved in the arachidonic acid (ARA) cascade: group 6 iPLA_2_ (Rn00588064_m1) (30), group 2a sPLA_2_ (Rn00580999_m1) (30), group 4 cPLA_2_ (Rn00591916_m1) (30), and COX-2 (Rn00568225_m1) (30); genes involved in neuroplasticity: brain-derived neurotrophic factor (Rn02531967_s1) (31), transthyretin (Rn00562124_m1) (32), T-cell intracellular antigen 1 (Rn01420836_m1) (32), and α-synuclein (Rn00569821_m1) (33); genes involved in the dopaminergic system: dopamine receptor D2 (Rn00561126_m1) (34), vesicular monoamine transporter 2 (Rn00564688_m1) (34), and tyrosine hydroxylase (Rn_00562500_m1) (34); genes involved in learning and memory: retinoic acid receptor α (Rn00580551_m1) (35), retnoid X receptor α (Rn00441185_m1) (35), retnoid X receptor β (Rn01399560_m1) (35), and peroxisome proliferator-activated receptor α (Rn00440945_m1) (35); genes involved in neurodegeneration and neuroinflammation: uncoupling protein 2 (Rn01754856_m1) (32), TNF-α receptor member 1a (Rn01492348m1) (33), heme oxygenase 1 (Rn01536933_m1) (36), and 15-lipoxygenase (Rn00696151_m1) (37); as well as epidermal growth factor receptor (Rn00580398_m1) (32) and prostaglandin E synthase 3 (Rn01529546_m1) (32). Endogenous controls measured included 18S RNA, phosphoglycerate kinase 1 (Rn01474011_g1) and β-actin (Rn00667869_m1). All genes were normalized to phosphoglycerate kinase 1. Similar results were found when normalizing to β-actin or 18S rRNA.

### Steady-state infusion study: surgery and ^2^H-ALA infusion

At 15 weeks postweaning, rats were subjected to surgery to implant a catheter into their jugular vein. The animals were anesthetized using isoflurane inhalation (5% induction, 1–3% maintenance). Before the incision was made, hair was shaved from the incision site and the site was sterilized with iodine and ethanol. A transverse incision was made anterior to the upper thorax. The jugular vein was located by blunt dissection. The vein was isolated and tied off. The vein was then nicked and a catheter (PE 50; Intramedic, Sparks, MD) with a 3.5 cm silastic tubing end (VWR, Mississauga, ON, Canada) was inserted into the vein. The catheter was secured using 3.0 silk sutures and a 16 gauge Angiocath (Becton Dickinson, Mississauga, ON, Canada) was used to guide the catheter subcutaneously to a site outside the body near the scapula. An incision was made at this site and the catheter was tucked beneath the skin at the incision site. The incision site was stapled to protect the catheter from the rats. The incision site on the chest was closed with 4.0 silk sutures and the rats were allowed to recover from the anesthetic under a heat lamp. Approximately 24 h after the surgery, the tail vein of the rats was cannulated with a 24 gauge Angiocath (Becton Dickinson). While the rats were restrained, the staple closing the incision site on the scapula was removed and the jugular vein catheter was connected to a longer polyethylene catheter. The rats were then placed in an infusion box that contained food, a chew toy, and bedding from the rat's cage. The tail vein catheter was then connected to an infusion line. Rats were able to move freely within the infusion box throughout the infusion.

Modified from the method of Rapoport, Igarashi, and Gao (38); 4.5 μmol/100 g body weight of ^2^H-ALA (^2^H_14_-ALA, purity >95% confirmed by GC-FID and GC-MS; Cayman Chemical, Ann Arbor, MI) was infused into the tail vein for 3 h. To prepare the infusate, a known amount of ^2^H-ALA was dissolved in 5 mM HEPES buffer (pH 7.4) containing 50 mg/ml fatty acid-free BSA. The infusate was mixed by sonication at 37°C. An infusion pump (Harvard Apparatus PHD 2000; Holliston, MA) was used to infuse 3.78 ml of tracer solution at a constant rate of 0.021 ml/min for 3 h. Immediately before the infusion and every 30 min during the infusion 0.2 ml of blood was drawn from the jugular vein. The jugular vein catheter was flushed with heparinized saline (5% by volume) after every blood draw to prevent coagulation in the line. After 180 min of the infusion, 1 ml of blood was drawn from the jugular vein and the animals were euthanized with a lethal injection of T-61 into the tail vein. All blood samples were centrifuged for 10 min (PC-100 microcentrifuge; Diamed, ON, Canada) and the plasma was collected and stored at −80°C.

### Steady-state infusion study: determination of plasma volume

Plasma volume was determined using the method of Schreihofer, Hair, and Stepp as modified by Gao et al.(24, 39). Briefly, a known amount of Evans Blue dye was injected into the tail veins of the rats. After 15 min, 1 ml of blood was drawn from the jugular vein, twice. The plasma was collected as described above, and 0.1 ml of plasma was diluted into 1 ml of saline. Absorbance at 604 nm was measured with a Nanodrop 1000 and by comparing the absorbance to a standard curve, the concentration of the dye was determined.

### Steady-state infusion study: plasma lipid extraction

Fifty microliters of plasma were added to a test tube which contained known amounts of unesterified 17:0 standard and di-17:0 phosphatidylcholine standard. Lipids were extracted from the plasma using the method of Folch, Lees, and Sloane Stanley (28), as described above.

### Steady-state infusion study: thin layer chromatography

Thin layer chromatography (TLC) was used to separate esterified and unesterified lipids. The TLE was dried under N_2_ and reconstituted in 250 μl chloroform. TLC plates (TLC silica gel 60, EMD) that were washed in chloroform and methanol (2:1) were activated by heat at 100°C for 1 h. TLE was loaded onto the TLC plates and the plates were run in heptane-diethyl ether-glacial acetic acid (60:40:2 v/v/v) alongside authentic standards (Nu-Check Prep). The plates were sprayed with 0.1% (w/v) 8-anilino-1-naphthalenesulfonic acid. Total phospholipid, unesterified fatty acid, triglyceride, and cholesteryl ester bands were identified under UV light by comparison to standards. Esterified lipid (phospholipid, triglyceride, and cholesteryl ester) bands were collected and transferred to a glass test tube. The unesterified fatty acid band was scraped off and transferred to a separate glass test tube.

### Steady-state infusion study: plasma lipid hydroxylation and esterification

Plasma samples taken at time 0 min of the infusion were transmethylated as described above and run on GC-FID. Unesterified fatty acids were extracted from the silica using chloroform:methanol:0.88% KCl (2:1:0.8) as described above. Esterified lipids were hydrolyzed with 1 ml of 10% methanoic KOH at 70°C for 1 h (24). After the reaction, 1 ml of concentrated HCl was added to the sample followed by 1 ml of dH_2_O. The fatty acids were extracted twice with 3 ml of hexane. The unesterified lipids (free fatty acids and hydrolyzed esterified fatty acids) were converted to fatty acid-pentafluorobenzyl (PFB) esters by following the method of Strife and Murphy (40). One hundred microliters of a mixture of pentafluorobenzylbromide-diisopropylamine-acetonitrile (10:100:1,000 by volume) was added to the samples, which were then shaken for 15 min. The PFB mixture was then evaporated under N_2_ gas and the fatty acid-PFB esters were reconstituted in 50 μl of hexane and run on the GC mass spectrometer.

### GC-MS

Fatty acid-PFB esters were analyzed using an Agilent 6890 series gas chromatograph (Agilent Technologies, Wilmington, DE) equipped with a DB-FFAP capillary column (30 m × 0.25 mm interior diameter, 0.25 mm film thickness; J and W Scientific, Folsom, CA) according to the method of Pawlosky, Sprecher, and Salem (41). Fatty acid-PFB esters dissolved in hexane were injected with a splitless injection technique. The GC oven temperature was programmed from 80°C to 185°C at 20°C/min then to 240°C at 10°C/min and held for 30 min. The injector and transfer line were maintained at 250°C and 280°C, respectively. The negative chemical ionization source temperature was 150°C. Methane was the ionization gas. Selected ion mode was used to analyze fatty acids using the mass-PFB ion for PFB derivatives. The *m/z* values for ^2^H-ALA and ^2^H-DHA were 291 and 337, respectively. Concentrations were determined by using calibration equations that relate the fatty acid peak area:standard peak area ratio to a fatty acid concentration.

### Brain DHA uptake study: ^14^C-DHA infusion

At 15 weeks postweaning, a surgery was performed to implant a catheter into the jugular vein (described above). Approximately 24 h after the surgery, the tail vein of the rats was cannulated and ^14^C-DHA (76 μCi per rat) was infused into the tail vein at a rate of 0.223(1 + e^−19.2^*^t^*) ml/min (*t* is infusion time in minutes) for 5 min. At approximately 0, 15, 30, 45, 90, 180, 240, and 300 s during the infusion, 200 μl of blood was drawn from the jugular vein and the plasma was extracted and stored at −80°C as described above. After 5 min of ^14^C-DHA infusion rats were euthanized by high-energy head-focused microwave fixation (13.5 kW for 1.6 s). The brains were then removed, dissected sagittally and stored at −80°C until analysis.

### Brain DHA uptake study: plasma radioactivity analysis

Lipids were extracted from 50 μl of plasma using the method of Folch, Lees, and Sloane Stanley (28), as described above. A known portion of the plasma TLE was then added to a scintillation vial with 5 ml of scintillation cocktail (GE Healthcare Life Sciences, Baie d'Urfe, QC, Canada). Radioactivity was measured using liquid scintillation counting to determine the plasma AUC for radioactivity. For plasma samples drawn at time 0 s of the infusion, the unesterified lipids were isolated by TLC and quantified using GC-FID (described above).

### Brain DHA uptake study: liquid scintillation counting

Radioactivity was quantified by a Packard TRI-CARB2900TR liquid scintillation analyzer (Packard, Meriden, CT) with a detector efficiency of 48.8%. Radioactivity was expressed in disintegrations per minute; then converted to nCi.

### Brain DHA uptake study: brain radioactivity analysis

Brain hemispheres were homogenized and lipids extracted as described above. A known portion of the TLE was then added to a scintillation tube with 5 ml of scintillation cocktail. Radioactivity was quantified using liquid scintillation counting.

### Gavage study: surgery and blood sampling

At 10 weeks postweaning rats were subjected to a surgery to implant a catheter into their carotid artery. The animals were anesthetized using isoflurane inhalation (5% induction, 1–3% maintenance). Before the incision was made, hair was shaved from the incision site and the site was sterilized with iodine and ethanol. A transverse incision was made anterior to the upper thorax. The carotid artery was located by blunt dissection. The artery was isolated and tied off. The artery was then nicked and a catheter (PE 50, Intramedic, USA) was inserted into the vessel. The catheter was secured using 3.0 silk sutures and a 16 gauge Angiocath (Becton Dickinson) was used to guide the catheter subcutaneously to a site outside the body near the scapula. The incision site on the chest of rats was closed with 4.0 silk sutures and the rat was then injected with 1ml of saline solution subcutaneously and allowed to recover from the anesthetic under a heat lamp. The day after the surgery, 200 μl of blood was drawn from the carotid artery catheter; the rat was then gavaged with 10 mg of ^2^H_5_-ALA that was dissolved in 1 ml of olive oil (Sigma-Aldrich Corporation, St. Louis, MO). To prepare the gavage solution, ^2^H_5_-ALA ethyl ester (Cambridge Isotope Laboratories Inc., Andover, MA), which was generously donated by Dr. Stephen Cunnane, was hydrolyzed with 10% methanolic KOH at 70°C for 1 h. Unesterified fatty acids were formed by adding 1 ml of HCl followed by 1 ml of dH_2_O. Lipids were extracted twice with 3 ml of hexane. The unesterified ^2^H_5_-ALA was then dried down and mixed with olive oil to make a solution that was 10 mg ^2^H_5_-ALA per ml of olive oil. Blood samples (200 μl) were drawn at 30, 60, 90, 120, 180, 240, 300, and 360 min after the gavage. Plasma was separated and stored in −80°C as described above. Lipids were extracted from 50 μl of plasma by the Folch method, and the TLE was hydrolyzed to create unesterified fatty acids (described above). Hydrolyzed TLE was stored in −80°C until analyzed with liquid chromatography-tandem mass spectrometry (LC-MS/MS)

### LC-MS/MS

Half of the TLE was evaporated under N_2_ gas and reconstituted in 100 μl of water/acetonitrile (80:20 v/v). Fatty acids were detected using an Agilent HPLC 1290 (Agilent Technologies, Santa Clara, CA) equipped with an Agilent Zorbax SB-phenyl column (3 × 50 mm, 3.5 μm; AgilentTechnologies). The initial HPLC conditions of elution were set at 500 μl/min gradient system consisting of (A) 50% water and (B) 50% acetonitrile. The gradient started with 50% (A) and 50% (B) and maintained for 1.5 min, increased to 100% (B) from 1.5 to 6 min, and maintained at 100% B for 4 min to complete the total run of 10 min. Mass spectrometry analyses were carried out on QTRAP 5500 triple quadruple mass spectrometer (AB SCIEX, Framingham, MA) in electrospray ionization negative ion mode. The source temperature was 600°C and the ion spray voltage was −4,500 eV. The optimized parameters were as follows: declustering potential, −40; entrance potential, −10; collision energy, −20; and collision cell exit potential, −11. Mass transitions for ^2^H_5_-ALA, ^2^H_5_-eicosapentaenoic acid (EPA), ^2^H_5_-docosapentaenoic acid (DPA)n-3, and ^2^H_5_-DHA were: *m/z* 282.2 to 59.0, *m/z* 306.2 to 262.2, *m/z* 334.2 to 290.2, and *m/z* 332.2 to 288.2, respectively. Concentration was quantified by comparing the peak area ratios (peak of interest:internal standard) and correcting for a response factor that was determined for each fatty acid of interest. Response factors were determined by analyzing a standard mixture of 100 ng/ml each of DHA, EPA, ALA, DPA, and ^2^H_8_-ARA by LC/MS/MS and comparing peak areas for each of the four fatty acids in relation to the peak area for ^2^H_8_-AA to generate response factors. The response factors were 10, 0.75, 0.25, and 0.75 for ALA, EPA, DPAn-3, and DHA, respectively.

### Balance study: equation

In the balance study, PUFA content in the whole body of animals at time 0 (21 days of age) and after 15 weeks (126 days of age) consuming the diets was determined. Because PUFAs cannot be synthesized de novo, it is possible to determine the accretion of a specific PUFA by subtracting the baseline amount from the amount of that PUFA in rats at 15 weeks postweaning. Using equation 1, it is then possible to determine the metabolic consumption of the dietary PUFAs (25).

(*Eq. 1*) $$\begin{array}{l} \mathrm{metabolic consumption}={\text{dietary consumption}}_{i}-{\mathrm{excretion}}_{i}\\ -\left({\mathrm{accretion}}_{i}+{\mathrm{accretion}}_{x}\right) \end{array}$$

where *i* refers to a PUFA consumed in the diet of the animals and *x* refers to a longer chain PUFA synthesized from *i.*

### Steady-state infusion study: equations

To determine the DHA synthesis rate (steady-state infusion study), appearance of ^2^H-DHA in the plasma-esterified pool was measured and fit to a Boltzmann sigmoidal curve ([^2^H-DHA] × plasma volume vs. time) using nonlinear curve (24) (Graphpad Prism Version 4.0, La Jolla, CA). At any point on this curve, the slope (*S*) will be determined by the ability of the body to synthesize ^2^H-DHA from ^2^H-ALA and the ability of the periphery to uptake ^2^H-DHA (equation 2).

(*Eq. 2*) $$S=k_{1,\text{DHA}}{{[}^{2}\text{H-ALA}]}_{\text{unesterified}}-k_{-1,\text{DHA}}{{[}^{2}\text{H-DHA}]}_{\text{esterified}}$$

where *k*_1,DHA_ is the steady-state synthesis-secretion coefficient for DHA, [^2^H-ALA]_unesterified_ is the plasma concentration of the infusate, *k*_−1,DHA_ is the disappearance coefficient for DHA, and [^2^H-DHA]_esterified_ is the concentration of DHA in the plasma that has been synthesized from the infusate, packaged into a lipoprotein and secreted into the plasma.

The maximum first derivative (*S*_max_) of this curve is assumed to be the time point when the uptake of esterified DHA from the periphery is negligible, i.e., 0 (equation 3).

(*Eq. 3*) $$S_{\max}=k_{1,\text{DHA}}{{[}^{2}\text{H-ALA}]}_{\text{unesterified}}-0$$

Therefore, the derivative at this point is equal to the rate of ^2^H-DHA synthesis. By correcting the *S*_max_ by the tracee:tracer ratio, the rats actual DHA synthesis is determined, *J*_syn,DHA_ (nmol/min) (24).

(*Eq. 4*) Jsyn,DHA=Smax[ALA]unesterified[H2-ALA]unesterified=k1,DHA[ALA]unesterified

### Brain DHA uptake study: equations

To determine the brain DHA uptake rate, the incorporation coefficient for DHA from the unesterified plasma pool into the brain total lipid pool is (26):

(*Eq. 5*) $$k^{*}=\frac{c_{\text{br}}^{*}\left(T\right)}{\int_{0}^{T}c_{\text{pl}}^{*}dt}$$

where $c_{\text{br}}^{*}\left(T\right)$ is the total brain radioactivity at the end of the infusion and $\int_{0}^{T}c_{\text{pl}}^{*}dt$ is the plasma radioactivity AUC.

Multiplying the incorporation coefficient by the concentration of plasma unesterified DHA (*c*_pl_) allows for the determination of the brain DHA uptake rate:

(*Eq. 6*) $$J_{\text{in}}=k^{*}c_{\text{pl}}$$

### Statistics

Mean lipid concentrations and accretions, as well as mean relative quantitation for genes and brain DHA uptake rates, were compared by one-way ANOVA using Tukey's test for multiple comparison (Graphpad Prism version 4.0). If variances were determined to be unequal, by Bartlett's test for equality of variances, then the Kruskal-Wallis test was used to compare the means followed by Dunn's test for multiple comparisons.

Mean plasma lipid concentrations were compared using the Kruskal-Wallis test followed by Dunn's test for multiple comparisons.

DHA synthesis rates between rats consuming the ALA and control diet was compared using Student's *t*-test due to the fact that DHA synthesis rates were not quantifiable in rats consuming the DHA diet.

## RESULTS

### Body weight and food intake: balance study

There were no statistical differences in body weight and food intake between rats consuming different diets throughout the 15 week feeding period. At 15 weeks, mean weights were 713 ± 9 g, 673 ± 25 g, and 724 ± 18 g in rats fed the control, ALA, and DHA diet, respectively. Average weekly food intake throughout the study was 190 ± 5 g, 175 ± 5 g, and 189 ± 7 g for the control, ALA, and DHA diet, respectively.

### Fecal excretion of PUFAs: balance study

On average, 0.27 ± 0.002%, 0.07 ± 0.02%, and 0.5 ± 0.05% of dietary ALA, DHA, and LNA, respectively, were excreted in the feces.

### Baseline PUFA concentrations: balance study

Total n-6 and n-3 PUFAs in 21-day-old rats were 1505 ± 35 μmol and 148 ± 4 μmol, respectively (supplementary Table I and **Table 2**). The majority of PUFAs were in the carcasses of the rats. LNA was the main n-6 PUFA at baseline 1,279 ± 30 μmol (data not shown). DHA and ALA were the most abundant n-3 PUFAs at baseline (55 ± 1 μmol and 41 ± 0.9 μmol, respectively, data not shown).

**TABLE 2.** Summary of n-3 PUFA Balance

Dietary Group	Control	ALA	DHA
Intake of n-3 PUFAs (μmol)	2560 ± 69*	21273 ± 661*	17301 ± 627**
Fecal excretion (μmol)	7 ± 0.2	57 ± 2	10 ± 0.4**
Body content of n-3 PUFAs (μmol)			
Day 0	139 ± 3	139 ± 3	139 ± 3
Day 105	380 ± 23^a^	2352 ± 166^b^	1607 ± 106^b^
Brain content of n-3 PUFAs (μmol)			
Day 0	10 ± 0.6	10 ± 0.6	10 ± 0.6
Day 105	9.7 ± 1^a^	14 ± 2^b^	15 ± 3^b^
Total accretion (μmol)			
ALA	47 ± 6^a^	1500 ± 100^b^	51 ± 7^a^
20:3n-3	84 ± 7^a^	110 ± 11^ab^	164 ± 21^b^
EPA	45 ± 3^a^	70 ± 10^b^	71 ± 8^b^
DPAn-3	5 ± 2^a^	90 ± 7^b^	66 ± 7^b^
DHA	60 ± 6^a^	440 ± 39^b^	1119 ± 62^c^
Total n-3 PUFA	240 ± 17^a^	2209 ± 107^b^	1472 ± 99^b^
Metabolic consumption of dietary PUFAs (μmol)	2317 ± 61^a^	19037 ± 617^b^	15783 ± 548^b,^**

Data are mean ± SEM. Different letters signify means are significantly different (*P* < 0.05) measured by one-way ANOVA followed by Tukey's test for multiple comparisons. n = 2 for day 0 measurements and n = 11 for day 105 measurements.

*Consumption of ALA.

**Refers to DHA.

### Final PUFA concentrations: balance study

**Fig. 1.**
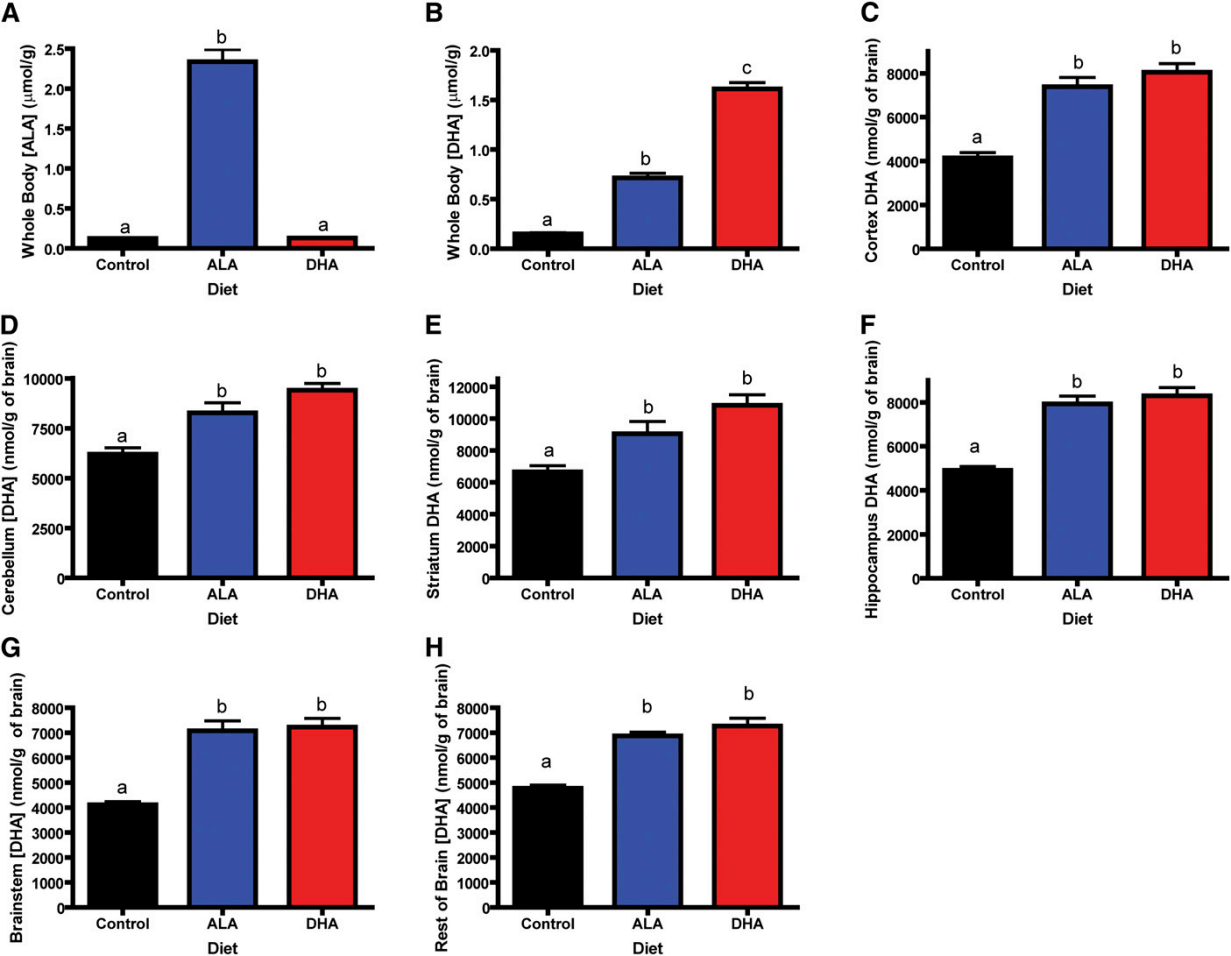
Whole body ALA and DHA concentrations and brain DHA concentrations in rats consuming the control, ALA, or DHA diet. A: Whole body ALA concentration (μmol/g) is highest in rats consuming ALA and is not different in animals consuming DHA or the control diet. B: Whole body DHA concentration (μmol/g) is highest in rats consuming DHA diet > ALA diet > control diet. Brain DHA concentrations (nmol/g) are not different in rats consuming the ALA and DHA diets but are significantly lower in rats consuming the control diet in the cortex (C), cerebellum (D), striatum (E), hippocampus (F), brainstem (G), and the rest of the brain (H). All data are mean ± SEM. Different letters signify the means are significantly different (*P* < 0.05) measured by one-way ANOVA followed by Tukey's multiple comparison test or Kruskal-Wallis test followed by Dunn's multiple comparison test (if variances were significantly different) (n = 11).

Concentrations of major n-3 PUFAs in the brain and whole body are shown in **Fig. 1**. Whole body ALA concentrations (Fig. 1A) were 30-fold greater in rats consuming the ALA diet compared with rats consuming the DHA and control diets (ALA diet > DHA diet = control diet, *P* < 0.05). Whole body DHA concentrations (Fig. 1B) were highest in rats consuming the DHA diet, followed by the rats consuming the ALA diet, and lowest in rats consuming the control diet (*P* < 0.05). Regional brain DHA concentrations did not differ significantly between rats consuming the DHA and ALA diets (Fig. 1C–H). However, DHA concentrations were significantly lower in rats consuming the control diet compared with rats consuming the ALA and DHA diets in all brain regions (*P* < 0.05). Total (body + brain) n-6 PUFA concentrations did not differ between rats consuming different diets (supplementary Table I). However, DPAn-6 in both the brain and body were highest in the rats consuming the control diet, followed by the rats consuming the ALA diet, and lowest in rats consuming the DHA diet (supplementary Tables I, II; *P* < 0.05). Whole body ARA concentrations only differed between rats consuming the control and DHA diets (2280 ± 98 μmol vs. 1688 ± 44 μmol respectively, *P* < 0.05). Brain ARA concentrations only differed in the brainstem of rats fed the control and DHA diets, while brainstem ARA concentrations for rats fed the ALA diet were similar to those of rats fed both diets (supplementary Tables III–VIII, *P* < 0.05).

### PUFA accretion: balance study

**Fig. 2.**
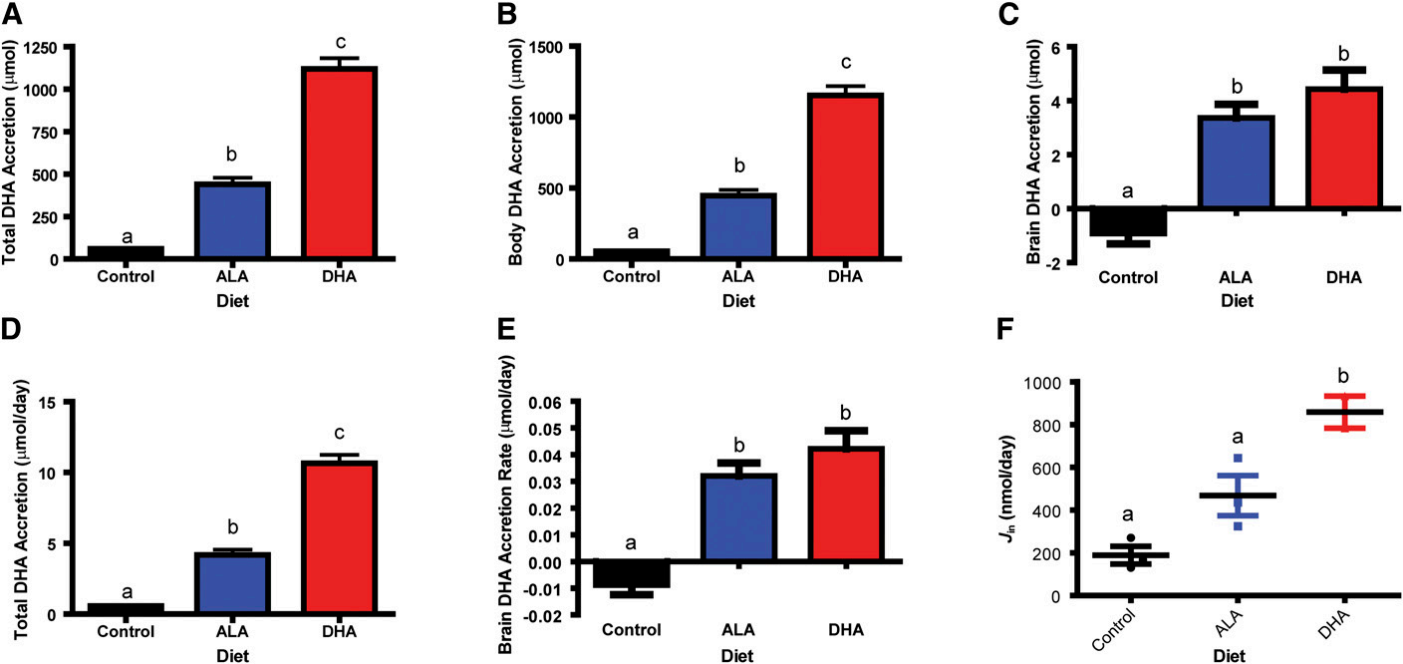
DHA kinetics. Total DHA accretion (body + brain DHA accretion) (A) and body DHA accretion (μmol) (B) was highest in rats consuming DHA > ALA > control diet (n = 11). C: Brain DHA accretion (μmol) was significantly lower in rats consuming the control diet compared with the rats consuming the ALA and DHA diets. There was no difference in brain DHA accretion between rats consuming the ALA and DHA diets (n = 11). D: Total DHA accretion rate (μmol/day) in rats consuming the three diets. For rats consuming the ALA diet, this is the synthesis-accretion rate (4.2 μmol/day) (n = 11). E: Brain DHA accretion rate (μmol/day) of rats consuming the three diets. Rats consuming the DHA diet accreted 0.042 μmol DHA/day in their brains (n = 11). F: Brain DHA uptake rate (nmol/day) (n = 3 for the ALA and control groups and n = 2 for the DHA group). All data are mean ± SEM. Different letters signify the means are significantly different (*P* < 0.05) measured by one-way ANOVA followed by Tukey's multiple comparison test or Kruskal-Wallis test followed by Dunn's multiple comparison test (if variances were significantly different).

Table 2 and supplementary Table I summarize the n-3 and n-6 PUFA balance. Total and body DHA accretion (**Fig. 2A, B**) was highest in rats consuming the DHA diet, followed by rats consuming the ALA diet, followed by rats consuming the control diet (*P* < 0.05). Brain DHA accretion (Fig. 2C), however, did not differ in rats consuming the ALA and DHA diets but was significantly lower in rats consuming the control diet (*P* < 0.05). Rats consuming the ALA diet synthesized and accreted 4.2 ± 0.4 μmol/day of total DHA (Fig. 2D). The DHA uptake and accretion rate into the brain for rats consuming the DHA diet was 42 ± 7 nmol/day (Fig. 2E).

### Gene expression: balance study

**Fig. 3.**
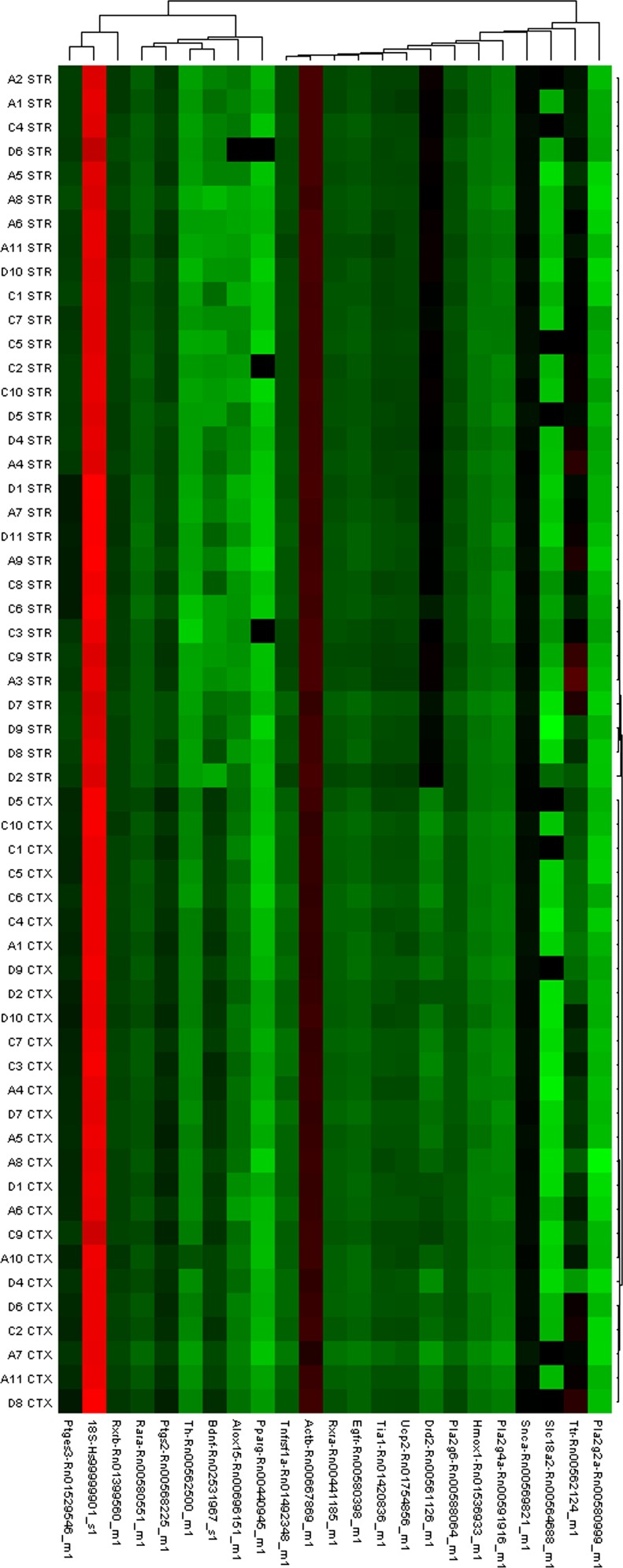
Heat map depicting gene expression in cortex and striatum brain samples. The figure illustrates that there are no differences within brain regions for any of the measured genes (average linkage and Pearson's distance metric). The clustering of all the cortex (CTX) samples and all the striatum (STR) samples together indicates that samples of the same brain region are highly correlated. There is also a clear difference in dopamine receptor D2 indicating brain regions were properly dissected as there was less expression of this receptor in the cortex relative to the striatum. Expression profiles are illustrated as ΔCt values with red indicating higher expression and green indicating lower expression compared with a striatal sample from a rat fed the ALA diet. Dendrograms indicate the correlation between groups of samples and genes. Samples are in columns and transcripts in rows. Gene names are available in the Materials and Methods section (n = 10).

Relative gene expression of 21 genes was measured in all six brain regions. A heat map representing gene expression in the cortex and striatum illustrates regional differences in gene expression (**Fig. 3**). While there were strong effects of brain region, diet did not have an effect on gene expression for most of the measured genes. There were only 11 differences in gene expression out of 378 comparisons. A full list of relative gene expression of all the genes measured is available in supplementary Tables IX–XIV. Due to the high number of comparisons, it is not possible to rule out these differences as a chance finding.

### Plasma volume: steady-state infusion study

Mean plasma volume (V_plasma_) was measured to be 37 ± 1.6 ml/kg which agrees with a previous report that measured plasma volume in Long Evans rats (42).

### Plasma concentrations: steady-state infusion study

Unesterified ALA in the plasma was higher in rats consuming the ALA diet compared with rats consuming the control diet (supplementary Table XV, *P* < 0.05) but did not differ from rats consuming the DHA diet (4 ± 0.9 nmol/ml, *P* > 0.05). Rats consuming the DHA diet had significantly higher plasma unesterified DHA levels than rats consuming the control diet (65 ± 21 nmol/ml vs. 1 ± 0.3 nmol/ml, *P* < 0.05). Rats consuming the ALA diet did not have statistically different concentrations of plasma unesterified DHA compared with rats consuming the DHA or control diets (4 ± 0.9 nmol/ml, *P* > 0.05). Plasma esterified ALA and DHA followed the same pattern as plasma unesterified ALA and DHA (supplementary Table XVI).

### DHA synthesis: steady-state infusion study

**Fig. 4.**
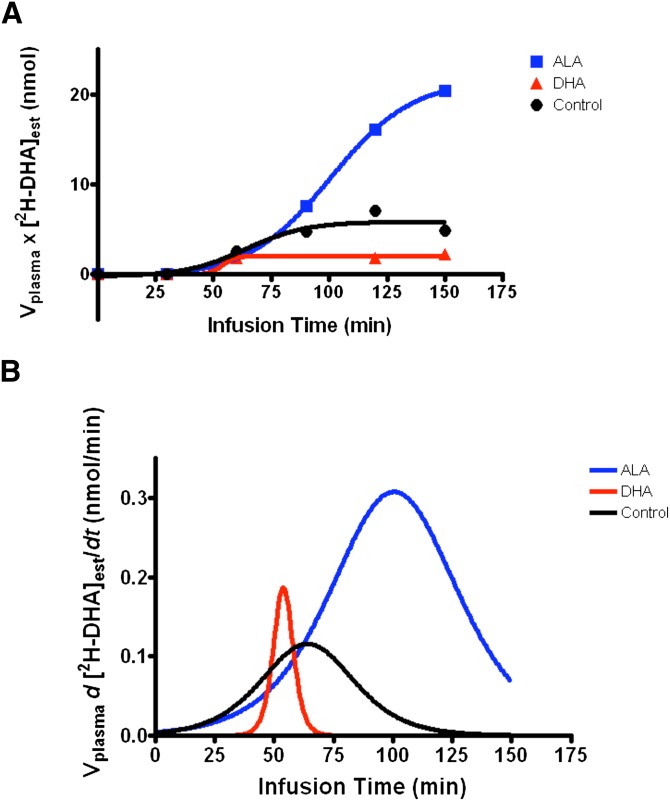
Example of infusion curves for a rat from each diet group. A: Plasma volume (ml) (V_plasma_) × concentration of esterified ^2^H-DHA (nmol) ([^2^H-DHA]_est_) plotted against time (min) and fit to a sigmoidal curve. This figure shows the resulting infusion curve for one rat from each dietary group that was infused with unesterified ^2^H-ALA for 3 h. B: First derivatives of the curves from (A). The maximum first derivative is used to determine the DHA synthesis rate. For the rat consuming DHA there was no increase in esterified ^2^H-DHA throughout the infusion resulting in an artificially high maximum first derivative, therefore, the DHA synthesis rate in this rat was deemed unquantifiable.

Curves in **Fig. 4A** show V_plasma_ × [^2^H-DHA]_esterified_ plotted versus time for three rats (one per dietary group). Due to the fact that [^2^H-DHA]_esterified_ did not increase throughout the infusion in rats consuming the DHA diet, and thus, the data did not fit to a sigmoidal curve; a DHA synthesis rate was not calculated in these animals. Mean infusion parameters are presented in **Table 3**. Mean [^2^H-ALA]_unesterified_ was 2.5 ± 0.5 nmol/ml and 10.5 ± 4.5 nmol/ml in the ALA diet and control diet groups, respectively. Mean *S*_max_ for rats consuming the ALA diet was 0.339 ± 0.135 nmol/min and 0.089 ± 0.023 nmol/min in rats consuming the control diet (*P* = 0.12). Corresponding daily DHA synthesis rates were 1452 ± 395 nmol/day and 45 ± 19 nmol/day in rats consuming the ALA and control diets, respectively (*P* < 0.05, Table 3).

**TABLE 3.** Parameters for synthesis-secretion of DHA in rats consuming the control, ALA, and DHA diet for 15 weeks

Diet	*S*_max,_*_i_* (nmol/min)	*k*_1,DHA_ (ml/min)	*J*_syn_ (nmol/min)	Daily Secretion Rate (nmol/day)	*F*_DHA_ (/min)	*t*_1/2_ (days)
Control	0.089 ± 0.023	0.012 ± 0.006	0.031 ± 0.013	45 ± 19	2E-05 ± 5E-06	57 ± 29
ALA	0.339 ± 0.135	0.131 ± 0.04*	1.008 ± 0.274*	1452 ± 395*	2E-04 ± 4E-05*	3 ± 1*
DHA	ND	ND	ND	ND	ND	ND

Data are mean ± SEM (n = 4, independent samples per group). ND, not determinable. *S*_max,_*_i_*, maximum first derivative; *k*_1,DHA_, synthesis-secretion coefficient for DHA synthesis from ALA; *J*_syn_, = synthesis rate of DHA from ALA; *F*_DHA_, turnover rate of esterified plasma DHA; *t*_1/2_, half-life of esterified plasma DHA.

*P < 0.05 versus control diet.

### Brain DHA uptake rate: brain DHA uptake study

There was no significant difference in k* between rats fed the different diets (5.9 ± 0.7 × 10^−4^ ml/s/g, 6.5 ± 1.9 × 10^−4^ ml/s/g, and 6.0 ± 1.2 × 10^−4^ ml/s/g for control, ALA, DHA diet, respectively). Brain DHA uptake rate was significantly higher in the DHA-fed rats versus the ALA and control-fed rats (858.6 ± 74.8 nmol/day > 467.6 ± 93.7 nmol/day = 189.1 ± 41.8 nmol/day respectively, *P* < 0.05). There were no statistical differences in brain DHA uptake rates between the ALA and control-fed rats (Fig. 2F).

### Appearance of ^2^H_5_-n-3 PUFAs: gavage study

**Fig. 5.**
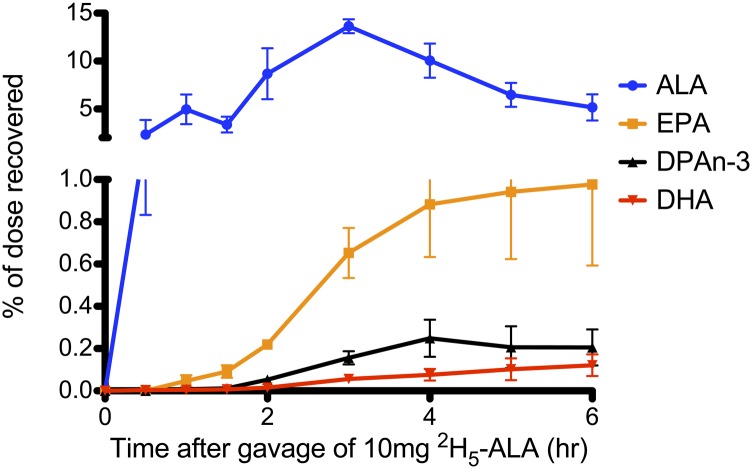
^2^H_5_-n-3 PUFA appearance in plasma of rats gavaged with 10 mg of ^2^H_5_-ALA. Data are expressed as mean percentage of dose recovered ± SEM at time points after the gavage. Different colored lines represent different ^2^H_5_-n-3 PUFAs. Data from this graph was used to model the methods used in humans to calculate DHA synthesis (n = 4).

**Figure 5** illustrates the appearance of ^2^H_5_-labeled n-3 PUFAs in the plasma of rats that were gavaged with 10 mg of ^2^H_5_-ALA. ALA was the first labeled n-3 PUFA to appear in the plasma and was consistently at the highest concentration. Labeled longer chain n-3 PUFAs, EPA, DPAn-3, and DHA, appeared in the plasma at later time points and concentrations of these PUFAs were lower compared with ALA.

## DISCUSSION

**Fig. 6.**
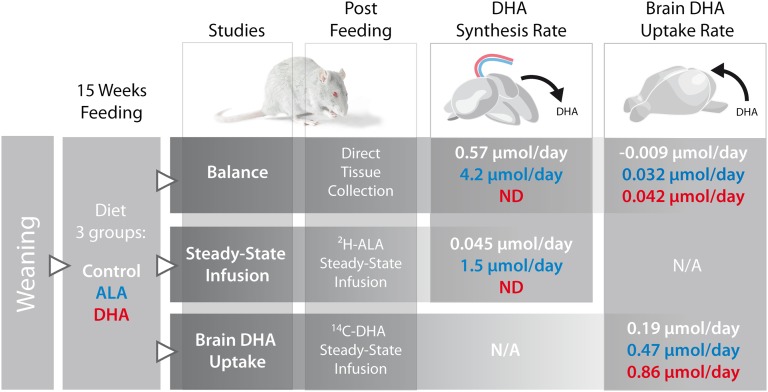
Summary of the methods and results. Three studies were performed in rats fed the control, ALA, or DHA diet from weaning for 15 weeks. After 15 weeks the brain and body were collected to perform the balance study. For the steady-state infusion study, a jugular vein and tail vein catheter were implanted into the rats and the rats were infused with ^2^H-ALA to measure whole body DHA synthesis from ALA in rats fed the three diets. For the brain DHA uptake study, tail vein and jugular vein catheters were implanted into the rats and ^14^C-DHA was infused to determine the brain DHA uptake rate in rats fed the three diets.

We showed that brain DHA levels in the adult rat can be maintained by dietary ALA just as well as by dietary DHA. This was supported by the finding that dietary ALA and DHA resulted in the same level and accretion of brain DHA after 15 weeks. From the 15 week balance study, the accretion of brain DHA in the ALA- and DHA-fed rats did not significantly differ, and equaled 0.032 ± 0.005 μmol per day and 0.042 ± 0.007 μmol per day, respectively (Fig. 2E). When we compare these daily brain accretion rates to the whole body DHA synthesis rate in the ALA-fed rats in the balance study, we see that DHA synthesis rates exceed brain uptake rates by 100-fold (Fig. 2D). To illustrate this, a summary of study designs and results is shown in **Fig. 6**. Using the steady-state infusion method, we estimated synthesis rates of DHA from ALA to be 1.5 and 0.045 μmol/day in animals consuming the ALA and control diets, respectively. These rates are lower, but in line with previously published estimates using the steady-state infusion technique (24, 43, 44). Differences could be due to the fact that we performed the steady-state infusion in rats fed a diet containing 2% of the total fatty acids as ALA, whereas Gao et al. (24) fed their animals a diet containing approximately 5% of the total fatty acids as ALA. The differences in synthesis rates could also be due to the differences in the age and strain of the rats. In particular, different rat strains are known to have different desaturase enzyme activity (45). Another important difference between our kinetic studies compared with the studies done by others is that our infusions were performed in completely free-living rats, 24 h after recovery from anesthesia (24, 46). Additionally, using a free-living infusion model, we determined the rate of DHA uptake from the unesterified plasma pool into the brain. Brain DHA uptake rates were between 189 and 618 nmol/day, which is similar to rates that have been previously published by others (46, 47).

Despite sizeable differences in n-3 PUFA concentrations and accretions in the bodies, we were unable to detect differences in brain DHA concentrations and accretions between rats consuming diets with DHA or ALA as the only n-3 PUFA source. In our balance study, we calculated a DHA synthesis rate of 4.2 μmol/day in animals fed the ALA diet. We also calculated, in rats consuming the DHA diet, a brain uptake and accretion rate for DHA of 42 nmol/day. Previous work in our lab found that mice fed DHA at a concentration of 2% of the fatty acids attained maximal DHA concentrations in the brain (48). Because rats fed the ALA diet were able to synthesize 100-fold more DHA than the amount of DHA accreted in the brains of rats consuming the DHA diet, and there were no significant differences in brain DHA concentrations or accretions in rats fed these two diets, it is likely that rats fed the ALA diet were able to synthesize sufficient DHA to maintain brain DHA levels. The finding that brain DHA concentrations are not different between rats fed the ALA and DHA diets is in contrast to previously published work (49). Abedin et al. (49) reported in 1999 that DHA in the brain phosphatidylethanolamine fraction was higher in Guinea pigs fed a diet containing DHA as compared with a diet containing ALA. Our study differed from this work, however, in that we measured brain total lipids, not brain phospholipid fractions, and we fed our rats pure fatty acid ethyl esters, whereas Abedin et al. (49) used oil sources to formulate their diets. As such, the high DHA diet also contained ALA in the study by Abedin et al. (49).

Furthermore, the results of our kinetic studies support the idea that rats consuming the ALA diet were able to synthesize enough DHA to supply the brain. Using the steady-state infusion method, we calculated that rats consuming the ALA diet synthesized 1.5 μmol/day of DHA, which is about 3-fold higher than the amount of DHA these rats uptake into the brain (468 nmol/day) as calculated by a 5 min infusion of radiolabeled DHA. Also, DHA synthesis rates were higher in rats fed a diet containing ALA compared with rats fed a diet with no added n-3 PUFAs. This suggests that the ALA substrate was the rate-limiting factor for DHA synthesis from ALA. Contrary to others, we were unable to measure a DHA synthesis rate in rats fed a diet containing DHA (24). Our work indicates that 2% dietary DHA reduced DHA synthesis by greater than 30-fold (24). It has been previously reported that n-3 PUFA feeding downregulates the expression of the hepatic desaturase enzymes required for DHA synthesis from ALA (50).

Despite the similar brain DHA concentrations in rats fed the ALA and DHA diets, rats consuming DHA had an almost 2-fold greater uptake rate of DHA into the brain. This means that the brains of rats fed the DHA diet took up and metabolically consumed more DHA. Despite increased metabolism of DHA in the brain, there were no differences in gene expression between rats on either diet. It is conceivable that exposing these rats to a stressor (such as brain trauma, neuroinflammation, etc.) would result in differential gene expression in the brains of these rats. Therefore, future experiments should measure the effect of diet on brain gene expression in rats that have been exposed to stress.

The steady-state infusion method developed by Rapoport and Igarashi (51) in 2009 is an in vivo kinetic approach to measure DHA synthesis rates at a given time, whereas the balance method measures an average synthesis rate over the balance period. Using the steady-state infusion method, we calculated that rats consuming the ALA diet were synthesizing 1.5 μmol of DHA per day at 15 weeks postweaning; whereas using the balance method, we calculated that over the 15 weeks these rats synthesized, on average, 4.2 μmol of DHA per day. One explanation as to why the balance method gives a higher DHA synthesis rate than the steady-state infusion method is because the balance study measures an average DHA synthesis rate over the 15 weeks. Therefore, included in the average rate is the growth and development period of the rat, when DHA synthesis rates are likely to be highest. In contrast, the steady-state infusion method measured DHA synthesis rates at the end of the 15 week feeding period. This rate is only a measure of the synthesis rate in these rats at this time, when the rats are older and when the DHA synthesis rate is also likely low. It has been shown previously that the DHA synthesis rate decreases with age in rats (44).

Using the balance method to calculate DHA synthesis rates is advantageous because it measures DHA in all body pools to determine how much DHA was synthesized and accreted. This method is limited, however, because it cannot determine how much DHA was synthesized and then metabolically consumed. Therefore this method likely underestimates the actual DHA synthesis rate. While the steady-state infusion method only measures the DHA in the plasma pool, this is unlikely to be a limitation because the method utilizes kinetic modeling to calculate the actual DHA synthesis rate. The steady-state infusion method is advantageous because it measures a DHA synthesis rate in conditions where the substrate may be rate limiting, which can be different from in vitro kinetic models where the substrate is assumed to not be rate limiting. More importantly, the steady-state infusion method may be advantageous to the oral administration method that is performed in humans to measure DHA synthesis. When ALA is consumed orally, the majority will be β-oxidized or stored in the adipose. Our study, as well as other balance studies, found that the majority of ALA intake is metabolically consumed and not accumulated in the tissues (25, 52). In our study approximately 90% of dietary ALA was metabolically consumed. While we did not measure adipose composition, other balance studies have found the majority of PUFAs that are not metabolically consumed are stored in the adipose (25). These findings are in agreement with oral tracer administration studies performed in humans (15). This is problematic because tracer that is stored in the adipose would not be available for DHA synthesis in the liver over the duration of the study. The steady-state infusion method eliminates this problem because the tracer is infused in a way that it achieves a steady-state level in the plasma despite uptake into the adipose.

It is generally accepted that the rat can synthesize more DHA than the human; however, the methods used to measure DHA synthesis in the human have not been validated in the rat. In humans, DHA synthesis is measured by administering an oral bolus of labeled ALA and measuring the appearance of labeled DHA in plasma. These calculations can be applied to the data from our gavage study to mimic the “human” method for determining DHA synthesis. When we applied three of the calculations previously performed in humans (12, 15, 53) to our data in rats, we obtained three different values for DHA synthesis. When using the calculation of McCloy et al. (15), mean AUC for ^2^H_5_-DHA in our rats was 0.31% of dose, which was in line with the value previously published in humans (0.99% of dose, corrected for plasma volume assuming plasma volume equals 4.5% of body weight). When we applied the calculation of Emken, Adlof, and Gulley (12) to our data in rats, the rats had lower average percent conversion to DHA compared with previously published values in humans (0.64 vs. 3.8%, respectively). Finally, when we used the calculation of Gillingham et al. (53), we found that the value of apparent conversion to DHA in the rat was comparable to that found in humans (0.12% of dose recovered as DHA in rat vs. 0.19% of dose recovered as DHA in human). Therefore, the results from the oral gavage study are inconsistent with the belief that the rat is more efficient at synthesizing DHA than the human. Also, the fact that applying different kinetic calculations to the same data gave markedly different results for DHA synthesis indicates that the calculations used to measure DHA synthesis are inconsistent and should be used largely to compare relative DHA synthesis between experimental groups within a study (22). While pilot data indicated that labeled DHA peaked within 6 h of an oral gavage in a rat, if this study was extended beyond 6 h, the DHA synthesis rates measured could be higher. However, we do not expect that extending this study beyond 6 h would increase synthesis rates enough to change our conclusions.

This study had several limitations. First, with respect to the gene expression data, we analyzed different sub-regions of the brain than others. For example, many studies analyzing expression of genes involved in the ARA cascade focused on the frontal cortex, whereas our study only investigated gene expression in the cortex, which could partly explain why we were unable to reproduce previously published findings (30, 31). This study was also limited by the 15 week feeding period. As all experiments were conducted on the rats at 15 weeks postweaning, we cannot draw conclusions about outcomes during the growth and development phase of the rat's life, when brain accretion peaks. It is possible that differences in brain DHA concentrations were more pronounced during adolescence and started to equilibrate in adulthood. In fact, it has been reported that infants who were breastfed had higher brain DHA concentrations than those fed formula, therefore, the results from our study do not apply to infants (54). Also, heparin was used as an anti-coagulant for our infusion studies. Heparin is known to activate lipoprotein lipase and may have contributed to the large variability in plasma unesterified fatty acid concentrations (55, 56). However, the effect of heparin is likely an overestimation of the rate of DHA uptake into the brain. Another limitation to this study was the use of pure fatty acid ethyl esters. Pure oil sources were used so that the effect of n-3 PUFAs could be compared directly (ALA vs. DHA). However, the use of pure oil sources limited the applicability of the study to free-living situations because n-3 PUFAs are consumed from food or oil sources, not as pure fatty acids, and most diets that contain DHA also contain ALA.

This study showed that despite large differences in fatty acid accumulation in the body, rats fed a diet containing DHA or ALA making up 2% of the fatty acids did not have differences in brain DHA accumulation. Using in vivo kinetic approaches, we were able to determine that animals consuming the ALA diet synthesized DHA at rates that exceed the rate of DHA uptake from the plasma into the brain. Importantly, rats consuming the ALA diet had a lower uptake rate of DHA into the brain than rats consuming DHA. As the uptake rate of DHA into the brain has been shown to match rates of brain DHA metabolism (27), it is likely that decreased brain DHA metabolism, in combination with an increased rate of DHA synthesis from ALA, is the reason that brain DHA accretion in rats fed the ALA diet did not differ from the rats fed the DHA diet. The overall results from this study indicate that DHA synthesis from ALA in the rat may be sufficient to maintain brain DHA concentrations in the absence of dietary DHA consumption. Importantly, the steady-state infusion method can be used in humans to calculate an actual DHA synthesis rate that can be compared with brain DHA uptake rates measured in humans with positron emission tomography scanning (57).

## Supplementary Material

Supplemental Data
