# Supplementary material for: Whole body synthesis rates of DHA from α-linolenic acid are greater than brain DHA accretion and uptake rates in adult rats
Source: J Lipid Res. 2014 Jan;55(1):62–74. doi: 10.1194/jlr.M042275 (PMC3927474; doi:10.1194/jlr.M042275)
Supplement: Supplemental Data [file supp_55_1_62__index.html]

Whole body synthesis rates of docosahexaenoic acid (DHA) from α-linolenic acid are greater than brain DHA accretion and uptake rates in adult rats. — Whole body synthesis rates of DHA from α-linolenic acid are greater than brain DHA accretion and uptake rates in adult rats — Supplemental Data 

# Whole body synthesis rates of DHA from α-linolenic acid are greater than brain DHA accretion and uptake rates in adult rats

## Supplemental Data

**Files in this Data Supplement:**

- Supplementary Table 1 - Supplementary Table 1: Summary of n-6 PUFA balance
- Supplementary Table 10 - Cortex gene expression in rats fed the control, ALA and DHA diet for 15 weeks
- Supplementary Table 11 - Brainstem gene expression in rats fed the control, ALA and DHA diet for 15 weeks
- Supplementary Table 12 - Hippocampus gene expression in rats fed the control, ALA and DHA diet for 15 weeks
- Supplementary Table 13 - Striatum gene expression in rats fed the control, ALA and DHA diet for 15 weeks
- Supplementary Table 14 - Rest of brain gene expression in rats fed the control, ALA and DHA diet for 15 weeks
- Supplementary Table 15 - Plasma unesterified fatty acid concentrations for rats fed the control, ALA or DHA diet for 15 weeks
- Supplementary Table 16 - Plasma esterified fatty acid concentrations for rats fed the control, ALA or DHA diet for 15 weeks
- Supplementary Table 2 - Whole body fatty acids for rats fed the control, ALA or DHA diet for 15 weeks
- Supplementary Table 3 - Cortex fatty acid concentrations for rats fed the control, ALA and DHA diets for 15 weeks
- Supplementary Table 4 - Cerebellum fatty acid concentrations for rats fed the control, ALA or DHA diet for 15 weeks
- Supplementary Table 5 - Striatum fatty acid concentrations for rats fed the control, ALA or DHA diet for 15 weeks
- Supplementary Table 6 - Hippocampus fatty acid concentrations for rats fed the control, ALA or DHA diet for 15 weeks
- Supplementary Table 7 - Brain stem fatty acid concentrations for rats fed the control, ALA or DHA diet for 15 weeks
- Supplementary Table 8 - Rest of brain fatty acid concentrations for rats fed the control, ALA or DHA diet for 15 weeks
- Supplementary Table 9 - Cerebellum gene expression in rats fed the control, ALA and DHA diet for 15 weeks
