# Supplementary material for: Whole body synthesis rates of DHA from α-linolenic acid are greater than brain DHA accretion and uptake rates in adult rats
Source: J Lipid Res. 2014 Jan;55(1):62–74. doi: 10.1194/jlr.M042275 (PMC3927474; doi:10.1194/jlr.M042275)
Supplement: Supplemental Data [file supp_M042275_jlr.M042275-1.pdf]

Supplementary Table 1: Summary of n-6 PUFA balance

Dietary Group:	Control (n=10)			ALA (n=10)			DHA (n=10)		
Intake of LNA (μmol)	190055	±	5097	175479	±	5456	189164	±	6855
Fecal Excretion of LNA (μmol)	950	±	25	877	±	27	946	±	34
Body Content of n-6 PUFA (μmol)									
Day 0	1491	±	35	1491	±	35	1491	±	35
Day 105	35231	±	1639	29170	±	2846	32391	±	2116
Brain Content of n-6 PUFA (μmol)									
Day 0	14	±	0.6	14	±	0.6	14	±	0.6
Day 105	24	±	0.9	20	±	0.9	17	±	0.9
Total Accretion (μmol)									
LNA	30313	±	2029	25157	±	2808	28810	±	3054
18:3n-6	57	±	5	52	±	5	41	±	5
20:2n-6	181	±	19	169	±	14	167	±	17
ARA	2132	±	97[a]	1806	±	93[ab]	1539	±	85[b]
22:4n-6	188	±	13[a]	138	±	12[a]	97	±	12[b]
DPAn-6	751	±	16[a]	168	±	9[b]	67	±	13[c]
Total	33621	±	1631	27489	±	2843	30720	±	2111
Metabolic consumption of dietary PUFA (μmol)	155702	±	4279	147161	±	5213	157517	±	5519

Data are means ± SEM, different letters signify means are significantly different ($p<0.05$) measured by One-Way ANOVA followed by Tukey's test for multiple comparisons
