# Supplementary material for: Whole body synthesis rates of DHA from α-linolenic acid are greater than brain DHA accretion and uptake rates in adult rats
Source: J Lipid Res. 2014 Jan;55(1):62–74. doi: 10.1194/jlr.M042275 (PMC3927474; doi:10.1194/jlr.M042275)
Supplement: Supplemental Data [file supp_M042275_jlr.M042275-10.pdf]

Supplementary Table 10. Cortex gene expression in rats fed the control, ALA and DHA diet for 15 weeks.

Dietary Group	Control	ALA	DHA
b actin	1 ± 0.15	1.05 ± 0.16	1.05 ± 0.22
15 LOX	1 ± 0.28	0.77 ± 0.28	1.04 ± 0.40
BDNF	1 ± 0.33	1.03 ± 0.33	1.16 ± 0.24
DR D2	1 ± 1.48	1.11 ± 1.01	1.12 ± 0.82
EGFR	1 ± 0.21	0.94 ± 0.17	0.99 ± 0.21
HO1	1 ± 0.18	1.05 ± 0.24	1.11 ± 0.26
spla2	1 ± 0.56	0.85 ± 0.49	1.17 ± 0.58
cPLA2	1 ± 0.20	0.93 ± 0.19	1.11 ± 0.30
iPLA2	1 ± 0.17	0.92 ± 0.19	1.09 ± 0.21
PPARg	1 ± 0.57	1.21 ± 0.62	1.36 ± 0.59
PGES3	1 ± 0.32	0.91 ± 0.35	1.07 ± 0.42
COX 2	1 ± 0.26	1.17 ± 0.43	1.29 ± 0.40
RAR a	1 ± 0.39	1.03 ± 0.41	1.20 ± 0.30
RXR a	1 ± 0.19	1.02 ± 0.18	1.03 ± 0.21
RXR b	1 ± 0.14	1.02 ± 0.28	1.05 ± 0.20
SNCa	1 ± 0.12	1.08 ± 0.15	1.15 ± 0.18
TH	1 ± 0.31	1.56 ± 1.70	1.11 ± 0.24
TIA1	1 ± 0.26	0.98 ± 0.23	1.06 ± 0.27
TNFaR1a	1 ± 0.28	1.11 ± 0.20	1.10 ± 0.25
TTR	1 ± 1.71	0.75 ± 0.97	1.49 ± 2.94
UCP2	1 ± 0.20	1.04 ± 0.19	1.02 ± 0.22

Data are expressed as mean RQ ± SD. Different letters signify the means are significantly different ($p<0.05$) measured by One-way ANOVA followed by Tukey's test for multiple comparisons or Kruskal-Wallis test followed by Dunn's multiple comparison test (if variances were significantly different).
