# Supplementary material for: Whole body synthesis rates of DHA from α-linolenic acid are greater than brain DHA accretion and uptake rates in adult rats
Source: J Lipid Res. 2014 Jan;55(1):62–74. doi: 10.1194/jlr.M042275 (PMC3927474; doi:10.1194/jlr.M042275)
Supplement: Supplemental Data [file supp_M042275_jlr.M042275-11.pdf]

**Supplementary Table 11. Brainstem gene expression in rats fed the control, ALA and DHA diet for 15 weeks.**

| Dietary Group | Control | | | ALA | | | DHA | | |
|---|---|---|---|---|---|---|---|---|---|
| b-actin | 1 | ± | 0.21 | 1.11 | ± | 0.27 | 1.10 | ± | 0.35 |
| 15 LOX | 1 | ± | 0.49[ab] | 0.91 | ± | 0.59[a] | 1.68 | ± | 0.88[b] |
| BDNF | 1 | ± | 0.38 | 0.72 | ± | 0.28 | 1.19 | ± | 0.72 |
| DR D2 | 1 | ± | 0.32 | 0.96 | ± | 0.20 | 0.88 | ± | 0.39 |
| EGFR | 1 | ± | 0.24 | 1.05 | ± | 0.23 | 1.14 | ± | 0.63 |
| HO1 | 1 | ± | 0.16 | 1.01 | ± | 0.19 | 0.99 | ± | 0.21 |
| sPLA2 | 1 | ± | 0.71 | 0.72 | ± | 0.67 | 1.10 | ± | 0.77 |
| cPLA2 | 1 | ± | 0.21 | 0.98 | ± | 0.19 | 6.22 | ± | 16.96 |
| iPLA2 | 1 | ± | 0.24 | 0.89 | ± | 0.20 | 1.10 | ± | 0.38 |
| PPARg | 1 | ± | 0.43 | 0.91 | ± | 0.36 | 1.11 | ± | 0.60 |
| PGES3 | 1 | ± | 0.19 | 1.14 | ± | 0.38 | 1.10 | ± | 0.27 |
| COX 2 | 1 | ± | 0.31 | 0.87 | ± | 0.30 | 3.34 | ± | 6.43 |
| RARa | 1 | ± | 0.21 | 1.00 | ± | 0.38 | 1.52 | ± | 0.96 |
| RXRa | 1 | ± | 0.18 | 0.99 | ± | 0.16 | 1.02 | ± | 0.22 |
| RXRb | 1 | ± | 0.26 | 1.15 | ± | 0.19 | 1.29 | ± | 0.44 |
| VMAT2 | 1 | ± | 0.35 | 1.34 | ± | 0.73 | 9.94 | ± | 27.63 |
| SNCa | 1 | ± | 0.23[ab] | 0.95 | ± | 0.18[a] | 2.25 | ± | 3.24[b] |
| TH | 1 | ± | 0.34 | 1.24 | ± | 0.64 | 0.98 | ± | 0.76 |
| TIA1 | 1 | ± | 0.22[ab] | 0.86 | ± | 0.30[a] | 1.61 | ± | 1.34[b] |
| TNFaR1a | 1 | ± | 0.24 | 1.30 | ± | 0.63 | 1.21 | ± | 0.44 |
| TTR | 1 | ± | 2.01 | 0.93 | ± | 1.42 | 0.46 | ± | 0.72 |
| UCP2 | 1 | ± | 0.19 | 1.05 | ± | 0.30 | 1.08 | ± | 0.30 |

Data are expressed as mean RQ ± SD. Different letters signify the means are significantly different ($p<0.05$) measured by One-way ANOVA followed by Tukey's test for multiple comparisons or Kruskal-Wallis test followed by Dunn's multiple comparison test (if variances were significantly different).
