# Supplementary material for: Whole body synthesis rates of DHA from α-linolenic acid are greater than brain DHA accretion and uptake rates in adult rats
Source: J Lipid Res. 2014 Jan;55(1):62–74. doi: 10.1194/jlr.M042275 (PMC3927474; doi:10.1194/jlr.M042275)
Supplement: Supplemental Data [file supp_M042275_jlr.M042275-12.pdf]

Supplementary Table 12. Hippocampus gene expression in rats fed the control, ALA and DHA diet for 15 weeks.

Dietary Group	Control			ALA			DHA		
B-actin	1	±	0.16	1.13	±	0.15	1.12	±	0.22
15 LOX	1	±	0.34	0.74	±	0.45	0.96	±	0.67
BDNF	1	±	0.18	1.03	±	0.22	1.13	±	0.16
DR D2	1	±	0.41	1.27	±	1.18	0.95	±	0.52
EGFR	1	±	0.09	1.10	±	0.19	1.04	±	0.22
HO1	1	±	0.13	1.01	±	0.23	1.03	±	0.24
sPLA2	1	±	0.38	1.08	±	0.61	0.93	±	0.50
cPLA2	1	±	0.36	1.10	±	0.24	0.98	±	0.23
iPLA2	1	±	0.10	1.02	±	0.15	1.05	±	0.18
PPARγ	1	±	0.73	0.47	±	0.33	0.47	±	0.22
PGES3	1	±	0.20	1.00	±	0.19	1.11	±	0.22
COX 2	1	±	0.34	1.15	±	0.25	1.12	±	0.25
RAR a	1	±	0.33	0.96	±	0.27	0.97	±	0.19
RXR a	1	±	0.17	1.08	±	0.19	1.10	±	0.14
RXR b	1	±	0.15	0.97	±	0.22	1.07	±	0.17
VMAT 2	1	±	1.43	0.84	±	0.70	1.26	±	1.73
SNCa	1	±	0.21	1.10	±	0.21	1.19	±	0.18
TH	1	±	0.32	1.15	±	0.70	1.01	±	0.41
TIA1	1	±	0.15	1.09	±	0.24	1.08	±	0.25
TNFa R 1a	1	±	0.20	1.14	±	0.22	0.99	±	0.26
TTR	1	±	1.91	6.67	±	10.18	2.40	±	2.88
UCP2	1	±	0.21	1.26	±	0.29	1.07	±	0.34

Data are expressed as mean RQ ± SD. Different letters signify the means are significantly different ($p<0.05$) measured by One-way ANOVA followed by Tukey's test for multiple comparisons or Kruskal-Wallis test followed by Dunn's multiple comparison test (if variances were significantly different).
