# Supplementary material for: Whole body synthesis rates of DHA from α-linolenic acid are greater than brain DHA accretion and uptake rates in adult rats
Source: J Lipid Res. 2014 Jan;55(1):62–74. doi: 10.1194/jlr.M042275 (PMC3927474; doi:10.1194/jlr.M042275)
Supplement: Supplemental Data [file supp_M042275_jlr.M042275-13.pdf]

Supplementary Table 13. Striatum gene expression in rats fed the control, ALA and DHA diet for 15 weeks.

Dietary Group	Control			ALA			DHA		
b actin	1	±	0.11	1.04	±	0.13	0.92	±	0.13
15 LOX	1	±	0.59	1.00	±	0.69	1.46	±	0.95
BDNF	1	±	1.10	1.01	±	0.80	2.10	±	1.98
DR D2	1	±	0.24	1.20	±	0.24	0.98	±	0.29
EGFR	1	±	0.27	1.00	±	0.24	0.82	±	0.22
HO1	1	±	0.18	1.09	±	0.21	0.94	±	0.12
sPLA2	1	±	0.41	0.46	±	0.39	0.68	±	0.65
cPLA2	1	±	0.39	1.04	±	0.19	0.92	±	0.21
iPLA2	1	±	0.14	0.96	±	0.18	0.90	±	0.17
PPARg	1	±	1.52	0.90	±	0.38	0.83	±	0.64
PGES3	1	±	0.65	0.95	±	0.69	0.92	±	0.64
COX 2	1	±	0.23[ab]	1.04	±	0.30[a]	0.75	±	0.17[b]
RARa	1	±	0.24	0.93	±	0.22	0.93	±	0.24
RXRa	1	±	0.22	1.14	±	0.14	1.02	±	0.21
RXRb	1	±	0.17	1.10	±	0.18	1.02	±	0.25
SNCa	1	±	0.13	1.05	±	0.14	0.97	±	0.13
TH	1	±	0.50	1.12	±	0.26	1.19	±	0.31
TIA1	1	±	0.14	1.01	±	0.24	0.89	±	0.19
TNFaR1a	1	±	0.22	0.98	±	0.22	0.88	±	0.26
TTR	1	±	1.37	2.37	±	4.92	0.66	±	0.77
UCP2	1	±	0.16	1.09	±	0.23	1.00	±	0.24

Data are expressed as mean RQ ± SD. Different letters signify the means are significantly different ($p<0.05$) measured by One-way ANOVA followed by Tukey’s test for multiple comparisons or Kruskal-Wallis test followed by Dunn’s multiple comparison test (if variances were significantly different).
