# Supplementary material for: Whole body synthesis rates of DHA from α-linolenic acid are greater than brain DHA accretion and uptake rates in adult rats
Source: J Lipid Res. 2014 Jan;55(1):62–74. doi: 10.1194/jlr.M042275 (PMC3927474; doi:10.1194/jlr.M042275)
Supplement: Supplemental Data [file supp_M042275_jlr.M042275-14.pdf]

Supplementary Table 14. Rest of brain gene expression in rats fed the control, ALA and DHA diet for 15 weeks.

Dietary Group	Control			ALA			DHA		
b actin	1	±	0.07	1.00	±	0.11	1.08	±	0.34
15 LOX	1	±	1.56	0.62	±	1.37	1.07	±	1.39
BDNF	1	±	0.31	0.99	±	0.46	0.99	±	0.25
DR D2	1	±	0.22	1.00	±	0.16	1.05	±	0.38
EGFR	1	±	0.10	1.06	±	0.14	1.08	±	0.36
HO1	1	±	0.15	1.00	±	0.13	1.04	±	0.27
sPLA2	1	±	0.43[ab]	0.67	±	0.36[a]	1.17	±	0.50[b]
cPLA2	1	±	0.15	0.85	±	0.21	0.99	±	0.32
iPLA2	1	±	0.15	0.95	±	0.15	1.10	±	0.43
PPARg	1	±	0.33	1.18	±	0.31	1.14	±	0.51
PGES3	1	±	0.19	0.99	±	0.20	1.14	±	0.26
COX 2	1	±	0.32	0.92	±	0.30	1.21	±	0.73
RAR a	1	±	0.08	0.91	±	0.14	1.09	±	0.46
RXR a	1	±	0.10	1.05	±	0.10	1.17	±	0.37
RXR b	1	±	0.13	0.91	±	0.07	1.12	±	0.58
VMAT2	1	±	0.30	1.12	±	0.22	1.08	±	0.38
SNCa	1	±	0.12	0.97	±	0.15	1.09	±	0.40
TH	1	±	0.18	1.09	±	0.20	1.18	±	0.67
TIA1	1	±	0.13	0.97	±	0.16	1.08	±	0.22
TNFaR1a	1	±	0.11	0.95	±	0.13	1.06	±	0.54
TTR	1	±	0.42	0.71	±	0.43	1.08	±	0.64
UCP2	1	±	0.10	0.97	±	0.15	1.20	±	0.81

Data are expressed as mean RQ ± SD. Different letters signify the means are significantly different ($p<0.05$) measured by One-way ANOVA followed by Tukey’s test for multiple comparisons or Kruskal-Wallis test followed by Dunn’s multiple comparison test (if variances were significantly different).
