# Supplementary material for: Whole body synthesis rates of DHA from α-linolenic acid are greater than brain DHA accretion and uptake rates in adult rats
Source: J Lipid Res. 2014 Jan;55(1):62–74. doi: 10.1194/jlr.M042275 (PMC3927474; doi:10.1194/jlr.M042275)
Supplement: Supplemental Data [file supp_M042275_jlr.M042275-15.pdf]

Supplementary Table 15. Plasma unesterified fatty acid concentrations for rats fed the control, ALA or DHA diet for 15 weeks

Fatty Acid	Control (n=3)	ALA (n=6)	DHA (n=5)
14:0	51 ± 15	15 ± 3	69 ± 24
16:0	287 ± 50[ab]	183 ± 33[a]	744 ± 138[b]
16:1n-7	35 ± 9[ab]	24 ± 5[a]	119 ± 26[b]
18:0	169 ± 10	128 ± 31	220 ± 28
18:1n-9	157 ± 48[ab]	104 ± 24[a]	500 ± 99[b]
18:1n-7	43 ± 15[ab]	29 ± 7[a]	118 ± 28[b]
18:2n-6	153 ± 39[ab]	106 ± 23[a]	408 ± 97.3[b]
18:3n-6	1 ± 0.4[ab]	1 ± 0.1[a]	2 ± 0.6[b]
18:3n-3	1 ± 0.3[a]	7 ± 1.6[b]	4 ± 0.9[ab]
20:0	2 ± 0.2	2 ± 0.5	3 ± 0.5
20:1n-9	3 ± 1.3	9 ± 5.3	7 ± 2
20:2n-6	2 ± 0.5[ab]	1.7 ± 0.4[a]	6 ± 2[b]
20:3n-3	7 ± 3	8 ± 3	15 ± 6
ARA (20:4n-6)	26 ± 7	15 ± 3.2	35 ± 8
EPA (20:5n-3)	5 ± 3	3 ± 1.7	5 ± 2
22:1n-9	48 ± 42	11 ± 4.3	8 ± 1
22:4n-6	4 ± 1	2.0 ± 0.4	5 ± 1.8
22:5n-6	5 ± 1.6[a]	1 ± 0.3[b]	2 ± 0.2[ab]
24:1n-9	3 ± 1	4 ± 2	6 ± 2
22:5n-3	3 ± 2.9	3 ± 2	5 ± 2
DHA (22:6n-3)	1 ± 0.3[a]	4 ± 0.9[ab]	65 ± 21[b]

Data shown are means +/- SEM and are expressed in nmol/ml of plasma. Different letters signify the means are significantly different ($p<0.05$) measured by Kruskal-Wallis test followed by Dunn's test for multiple comparisons.
