# Supplementary material for: Whole body synthesis rates of DHA from α-linolenic acid are greater than brain DHA accretion and uptake rates in adult rats
Source: J Lipid Res. 2014 Jan;55(1):62–74. doi: 10.1194/jlr.M042275 (PMC3927474; doi:10.1194/jlr.M042275)
Supplement: Supplemental Data [file supp_M042275_jlr.M042275-16.pdf]

Supplementary Table 16. Plasma esterified fatty acid concentrations for rats fed the control, ALA or DHA diet for 15 weeks

Fatty Acid	Control (n=3)			ALA (n=6)			DHA (n=5)		
14:0	80	±	21	85	±	31	52	±	9
16:0	880	±	568	1485	±	223	1550	±	252
16:1n-7	102	±	30	121	±	28	98	±	22
18:0	1715	±	450	1348	±	170	1617	±	368
18:1n-9	333	±	79	434	±	145	404	±	92
18:1n-7	164	±	53	203	±	32	139	±	27
18:2n-6	1065	±	275	971	±	114	1111	±	224
18:3n-6	11	±	3	7	±	1	6	±	2
18:3n-3	1	±	0.4[a]	12	±	7[b]	2	±	0.5[ab]
20:0	6	±	2	9	±	2	7	±	2
20:1n-9	8	±	5	16	±	7	8	±	4
20:2n-6	15	±	4	17	±	2	17	±	4
20:3n-3	58	±	21	60	±	8	118	±	26
ARA (20:4n-6)	2708	±	671	1978	±	259	1934	±	589
EPA (20:5n-3)	17	±	4	14	±	2	33	±	13
22:1n-9	25	±	14	24	±	10	16	±	4
22:4n-6	33	±	8[a]	18	±	4[ab]	9	±	2[b]
22:5n-6	230	±	46[a]	23	±	3[ab]	6	±	2[b]
24:1n-9	18	±	6	22	±	6	30	±	16
22:5n-3	4	±	1.1[a]	24	±	7[b]	15	±	4[ab]
DHA (22:6n-3)	73	±	19[a]	208	±	23[ab]	396	±	92[b]

Data shown are means +/- SEM and are expressed in nmol/ml of plasma. Different letters signify the means are significantly different ($p<0.05$) measured by Kruskal-Wallis test followed by Dunn’s test for multiple comparisons.
