# Supplementary material for: Whole body synthesis rates of DHA from α-linolenic acid are greater than brain DHA accretion and uptake rates in adult rats
Source: J Lipid Res. 2014 Jan;55(1):62–74. doi: 10.1194/jlr.M042275 (PMC3927474; doi:10.1194/jlr.M042275)
Supplement: Supplemental Data [file supp_M042275_jlr.M042275-2.pdf]

Supplementary Table 2: Whole body fatty acids for rats fed the control, ALA or DHA diet for 15 weeks

Fatty Acid	Control			ALA			DHA		
12:0	26353	±	1935	23532	±	2238	23339	±	2308
14:0	19223	±	1126	17606	±	1277	18211	±	1610
14:1n-7	1507	±	97	1331	±	109	1280	±	130
16:0	51757	±	3778	44604	±	3897	51092	±	5729
16:1n-7	18719	±	1581	16111	±	1454	17497	±	2287
18:0	6703	±	327	6101	±	299	6863	±	483
18:1n-9	45830	±	3338	38781	±	3325	40657	±	4800
18:1n-7	8998	±	674	7553	±	583	7340	±	739
18:2n-6	31605	±	1554	29104	±	1667	30102	±	2055
18:3n-6	73	±	5	67	±	5	56	±	5
18:3n-3	89	±	6 [a]	1557	±	99 [b]	93	±	7 [a]
20:1n-9	201	±	16	189	±	14	176	±	17
20:2	206	±	19	194	±	13	192	±	20
20:3n-3	112	±	7 [a]	137	±	11 [ab]	192	±	21 [b]
ARA (20:4n-6)	2280	±	98 [a]	1954	±	93 [ab]	1688	±	44 [b]
EPA (20:5n-3)	55	±	3 [a]	76	±	9 [b]	80	±	8 [b]
22:4n-6	205	±	16 [a]	155	±	12 [ab]	115	±	6 [b]
22:5n-6	533	±	21 [a]	124	±	6 [b]	52	±	3 [c]
22:5n-3	19	±	2 [a]	101	±	7 [b]	80	±	7 [b]
DHA (22:6n-3)	106	±	6 [a]	481	±	40 [b]	1160	±	63 [c]

Data are expressed in µmol, as mean +/- SEM. Different letters signify the means are significantly different ($p<0.05$) measured by One-way ANOVA followed by Tukey's test for multiple comparisons or Kruskal-Wallis test followed by Dunn's multiple comparison test (if variances were significantly different).
