# Supplementary material for: Whole body synthesis rates of DHA from α-linolenic acid are greater than brain DHA accretion and uptake rates in adult rats
Source: J Lipid Res. 2014 Jan;55(1):62–74. doi: 10.1194/jlr.M042275 (PMC3927474; doi:10.1194/jlr.M042275)
Supplement: Supplemental Data [file supp_M042275_jlr.M042275-3.pdf]

Supplementary Table 3. Cortex fatty acid concentrations for rats fed the control, ALA and DHA diets for 15 weeks

Fatty Acid	Control (n=11)			ALA (n=11)			DHA (n=11)		
14:0	186	±	6	176	±	5	191	±	6
16:0	18857	±	341	18295	±	216	18552	±	417
16:1n-7	313	±	33	351	±	34	361	±	35
18:0	17286	±	412	17115	±	223	17319	±	262
18:1n-9	14247	±	374	14618	±	330	14642	±	760
18:1 n-7	2744	±	62	2651	±	55	2670	±	61
18:2n-6	309	±	9 [a]	379	±	10 [b]	464	±	15 [c]
20:0	199	±	11	214	±	8	221	±	9
20:1n-9	801	±	27	846	±	33	848	±	73
20:2	81	±	4	74	±	8	85	±	3
20:3n-3	109	±	7 [a]	150	±	7 [b]	195	±	10 [c]
ARA (20:4n-6)	6440	±	222	6607	±	217	6344	±	197
EPA (20:5n-3)	196	±	9	212	±	10	223	±	14
22:1n-9	104	±	47	58	±	4	67	±	9
22:4n-6	1849	±	66 [a]	1745	±	59 [a]	1525	±	50 [b]
22:5 n-6	2719	±	124 [a]	275	±	18 [b]	76	±	6 [c]
24:1n-9	335	±	14 [a]	380	±	15 [ab]	403	±	20 [b]
22:5 n-3	20	±	2 [a]	57	±	4 [b]	68	±	4 [b]
DHA (22:6n-3)	3980	±	232 [a]	7096	±	407 [b]	7730	±	379 [b]

Data shown are means +/- SEM and are expressed in nmol/g of brain. Different letters signify the means are significantly different ($p<0.05$) measured by One-way ANOVA followed by Tukey’s test for multiple comparisons or Kruskal-Wallis test followed by Dunn’s multiple comparison test (if variances were significantly different).
