# Supplementary material for: Whole body synthesis rates of DHA from α-linolenic acid are greater than brain DHA accretion and uptake rates in adult rats
Source: J Lipid Res. 2014 Jan;55(1):62–74. doi: 10.1194/jlr.M042275 (PMC3927474; doi:10.1194/jlr.M042275)
Supplement: Supplemental Data [file supp_M042275_jlr.M042275-4.pdf]

Supplementary Table 4. Cerebellum fatty acid concentrations for rats fed the control, ALA or DHA diet for 15 weeks

Fatty Acid	Control (n=11)			ALA (n=11)			DHA (n=11)		
14:0	187	±	21	216	±	8	233	±	12
16:0	20542	±	538	20382	±	839	20035	±	246
16:1n-7	324	±	22	447	±	56	315	±	43
18:0	19206	±	625	19468	±	800	19542	±	444
18:1n-9	19343	±	757	20097	±	982	22498	±	1295
18:1n-7	4803	±	130	4770	±	181	4901	±	129
18:2n-6	681	±	39[a]	804	±	32[ab]	1025	±	74[b]
20:0	547	±	27	581	±	27	626	±	55
20:1n-9	2255	±	91[a]	2410	±	200[ab]	2838	±	247[b]
20:2n-6	212	±	10	228	±	7	251	±	15
20:3n-3	294	±	12[a]	320	±	14[a]	374	±	17[b]
ARA (20:4n-6)	6744	±	225	6304	±	255	5971	±	159
EPA (20:5n-3)	454	±	21	507	±	34	566	±	59
22:1n-9	196	±	12	200	±	14	199	±	12
22:4n-6	2469	±	85[a]	1899	±	67[b]	1598	±	58[c]
22:5n-6	3187	±	143[a]	214	±	15[b]	57	±	6[c]
24:1n-9	660	±	22	718	±	31	810	±	76
22:5n-3	26	±	1[a]	77	±	3[b]	99	±	5[b]
DHA (22:6n-3)	5958	±	309[a]	7943	±	483[b]	9032	±	328[b]

Data shown are means +/- SEM and are expressed in nmol/g of brain. Different letters signify the means are significantly different ($p<0.05$) measured by One-way ANOVA followed by Tukey's test for multiple comparisons or Kruskal-Wallis test followed by Dunn's multiple comparison test (if variances were significantly different).
