# Supplementary material for: Whole body synthesis rates of DHA from α-linolenic acid are greater than brain DHA accretion and uptake rates in adult rats
Source: J Lipid Res. 2014 Jan;55(1):62–74. doi: 10.1194/jlr.M042275 (PMC3927474; doi:10.1194/jlr.M042275)
Supplement: Supplemental Data [file supp_M042275_jlr.M042275-5.pdf]

Supplementary Table 5. Striatum fatty acid concentrations for rats fed the control, ALA or DHA diet for 15 weeks

Fatty Acid	Control (n=11)			ALA (n=11)			DHA (n=11)		
14:0	297	±	30	251	±	21	304	±	33
16:0	23013	±	769	21970	±	705	22887	±	678
16:1n-7	386	±	43	433	±	29	481	±	16
18:0	25623	±	910	25048	±	866	25703	±	540
18:1n-9	22958	±	1049	21774	±	984	23876	±	648
18:1n-7	4491	±	172	4189	±	167	4362	±	100
18:2n-6	492	±	24 [a]	561	±	27 [a]	684	±	25 [b]
20:0	471	±	35	407	±	24	437	±	18
20:1n-9	1882	±	120	1665	±	98	1873	±	98
20:2	127	±	9	111	±	8	123	±	7
20:3n-3	245	±	13 [a]	249	±	19 [a]	356	±	21 [b]
ARA (20:4n-6)	10044	±	511	9408	±	547	9410	±	389
EPA (20:5n-3)	578	±	50	479	±	32	499	±	48
22:1n-9	288	±	24 [a]	147	±	16 [b]	339	±	39 [a]
22:4n-6	2755	±	145 [a]	2387	±	145 [ab]	2196	±	95 [b]
22:5n-6	3356	±	180 [a]	331	±	24 [b]	89	±	6 [c]
24:1n-9	716	±	55	643	±	47	732	±	52
22:5n-3	15	±	2 [a]	54	±	3 [b]	60	±	5 [b]
DHA (22:6n-3)	6395	±	377 [a]	8698	±	742 [b]	10415	±	632 [b]

Data shown are means +/- SEM and are expressed in nmol/g of brain. Different letters signify the means are significantly different ($p<0.05$) measured by One-way ANOVA followed by Tukey’s test for multiple comparisons or Kruskal-Wallis test followed by Dunn’s multiple comparison test (if variances were significantly different).
