# Supplementary material for: Whole body synthesis rates of DHA from α-linolenic acid are greater than brain DHA accretion and uptake rates in adult rats
Source: J Lipid Res. 2014 Jan;55(1):62–74. doi: 10.1194/jlr.M042275 (PMC3927474; doi:10.1194/jlr.M042275)
Supplement: Supplemental Data [file supp_M042275_jlr.M042275-6.pdf]

Supplementary Table 6. Hippocampus fatty acid concentrations for rats fed the control, ALA or DHA diet for 15 weeks

Fatty Acid	Control (n=11)			ALA (n=11)			DHA (n=11)		
14:0	190	±	18	185	±	11	177	±	14
16:0	20923	±	482	20779	±	535	20484	±	601
16:1n-7	328	±	13	335	±	18	337	±	26
18:0	20404	±	466	20544	±	673	20431	±	609
18:1n-9	16727	±	622	17927	±	852	17851	±	518
18:1n-7	3065	±	94	3029	±	130	2923	±	72
18:2n-6	346	±	20 [a]	402	±	15 [a]	483	±	29 [b]
20:0	264	±	12	278	±	19	257	±	9
20:1n-9	888	±	54	915	±	67	891	±	35
20:2	99	±	4	108	±	7	110	±	4
20:3n-3	165	±	8 [a]	212	±	11 [b]	282	±	12 [c]
ARA (20:4n-6)	8312	±	191	9385	±	434	8674	±	401
EPA (20:5n-3)	267	±	16	263	±	33	207	±	32
22:1n-9	86	±	9	81	±	6	109	±	15
22:4n-6	2242	±	49	2325	±	116	2052	±	88
22:5n-6	3158	±	146 [a]	467	±	44 [b]	159	±	12 [c]
24:1n-9	382	±	18	448	±	39	420	±	22
22:5n-3	16	±	1 [a]	51	±	5 [b]	50	±	5 [b]
DHA (22:6n-3)	4715	±	163 [a]	7619	±	341 [b]	7970	±	362 [b]

Data shown are means +/- SEM and are expressed in nmol/g of brain. Different letters signify the means are significantly different ($p<0.05$) measured by One-way ANOVA followed by Tukey’s test for multiple comparisons or Kruskal-Wallis test followed by Dunn’s multiple comparison test (if variances were significantly different).
