# Supplementary material for: Whole body synthesis rates of DHA from α-linolenic acid are greater than brain DHA accretion and uptake rates in adult rats
Source: J Lipid Res. 2014 Jan;55(1):62–74. doi: 10.1194/jlr.M042275 (PMC3927474; doi:10.1194/jlr.M042275)
Supplement: Supplemental Data [file supp_M042275_jlr.M042275-7.pdf]

Supplementary Table 7. Brain stem fatty acid concentrations for rats fed the control, ALA or DHA diet for 15 weeks

Fatty Acid	Control (n=11)			ALA (n=11)			DHA (n=11)		
14:0	385	±	39	366	±	24	346	±	42
16:0	21680	±	926 [a]	20463	±	413 [ab]	20092	±	1106 [b]
16:1n-7	271	±	53	394	±	69	504	±	121
18:0	27867	±	1150	26673	±	533	27100	±	2378
18:1n-9	38598	±	2270	38569	±	972	37744	±	1713
18:1n-7	8318	±	278 [a]	7969	±	153 [ab]	7521	±	150 [b]
18:2n-6	696	±	43 [a]	835	±	33 [b]	1020	±	148 [b]
20:0	1926	±	118	1789	±	98	1695	±	56
20:1n-9	8088	±	363	7801	±	455	7599	±	217
20:2	468	±	30	418	±	18	409	±	15
20:3n-3	483	±	49	470	±	18	560	±	58
ARA (20:4n-6)	8755	±	339 [a]	7905	±	255 [ab]	7219	±	212 [b]
EPA (20:5n-3)	1677	±	139	1564	±	115	1432	±	96
22:1n-9	1324	±	237 [a]	1773	±	346 [a]	536	±	17 [b]
22:4n-6	4257	±	174 [a]	3083	±	121 [b]	2573	±	67 [b]
22:5n-6	3535	±	117 [a]	172	±	11 [b]	58	±	2 [c]
24:1n-9	2244	±	162	2230	±	156	2104	±	102
22:5n-3	41	±	9 [a]	76	±	4 [ab]	112	±	15 [b]
DHA (22:6n-3)	3956	±	112 [a]	6801	±	378 [b]	6945	±	315 [b]

Data shown are means +/- SEM and are expressed in nmol/g of brain. Different letters signify the means are significantly different ($p<0.05$) measured by One-way ANOVA followed by Tukey's test for multiple comparisons or Kruskal-Wallis test followed by Dunn's multiple comparison test (if variances were significantly different).
