# Supplementary material for: Whole body synthesis rates of DHA from α-linolenic acid are greater than brain DHA accretion and uptake rates in adult rats
Source: J Lipid Res. 2014 Jan;55(1):62–74. doi: 10.1194/jlr.M042275 (PMC3927474; doi:10.1194/jlr.M042275)
Supplement: Supplemental Data [file supp_M042275_jlr.M042275-8.pdf]

Supplementary Table 8. Rest of brain fatty acid concentrations for rats fed the control, ALA or DHA diet for 15 weeks

Fatty Acid	Control (n=11)			ALA (n=11)			DHA (n=11)		
14:0	176	±	6	189	±	12	164	±	4
16:0	17626	±	293	17550	±	294	17183	±	371
16:1n-7	229	±	31	329	±	38	229	±	33
18:0	18727	±	343	18500	±	398	18106	±	334
18:1n-9	18584	±	722	19280	±	661	19784	±	450
18:1n-7	3748	±	83	3718	±	109	3533	±	66
18:2n-6	389	±	15[a]	477	±	17[b]	556	±	16[c]
20:0	443	±	21	469	±	23	417	±	17
20:1n-9	1669	±	165	2074	±	82	1917	±	74
20:2	107	±	7	114	±	8	103	±	6
20:3n-3	212	±	6[a]	240	±	12[a]	297	±	11[b]
ARA (20:4n-6)	7227	±	182	6913	±	108	6653	±	247
EPA (20:5n-3)	412	±	17	442	±	20	410	±	15
22:1n-9	223	±	41	181	±	17	150	±	7
22:4n-6	2417	±	47[a]	2181	±	33[b]	1942	±	52[c]
22:5n-6	2481	±	68[a]	244	±	17[b]	92	±	7[c]
24:1n-9	594	±	24	672	±	30	621	±	29
22:5n-3	20	±	1[a]	59	±	2[b]	73	±	3[b]
DHA (22:6n-3)	4589	±	122[a]	6605	±	140[b]	6984	±	300[b]

Data shown are means +/- SEM and are expressed in nmol/g of brain. Different letters signify the means are significantly different ($p<0.05$) measured by One-way ANOVA followed by Tukey's test for multiple comparisons or Kruskal-Wallis test followed by Dunn's multiple comparison test (if variances were significantly different).
