# Supplementary material for: Whole body synthesis rates of DHA from α-linolenic acid are greater than brain DHA accretion and uptake rates in adult rats
Source: J Lipid Res. 2014 Jan;55(1):62–74. doi: 10.1194/jlr.M042275 (PMC3927474; doi:10.1194/jlr.M042275)
Supplement: Supplemental Data [file supp_M042275_jlr.M042275-9.pdf]

Supplementary Table 9. Cerebellum gene expression in rats fed the control, ALA and DHA diet for 15 weeks.

Dietary Group	Control			ALA			DHA		
b actin	1	±	0.12[ab]	1.10	±	0.08[a]	0.98	±	0.08[b]
15 LOX	1	±	0.61	0.63	±	0.30	1.35	±	1.00
BDNF	1	±	0.22	0.98	±	0.17	0.88	±	0.22
DR D2	1	±	0.21	1.13	±	0.34	1.09	±	0.35
EGFR	1	±	0.16[a]	1.26	±	0.10[b]	0.96	±	0.30[a]
HO1	1	±	0.17[a]	1.21	±	0.14[b]	0.99	±	0.22[a]
sPLA2	1	±	0.77	0.86	±	0.56	0.98	±	0.72
cPLA2	1	±	0.14	1.17	±	0.25	0.98	±	0.21
iPLA2	1	±	0.15	1.02	±	0.12	1.00	±	0.14
PPARg	1	±	0.13	1.17	±	0.24	1.07	±	0.23
PGES3	1	±	0.24	1.09	±	0.25	0.97	±	0.12
COX 2	1	±	0.33[a]	1.46	±	0.42[b]	1.41	±	0.32[ab]
RARa	1	±	0.23	1.07	±	0.19	1.08	±	0.18
RXRa	1	±	0.12[a]	1.15	±	0.12[b]	0.98	±	0.14[a]
RXRb	1	±	0.14	1.04	±	0.13	0.96	±	0.15
SNCa	1	±	0.14	0.96	±	0.11	0.84	±	0.21
TH	1	±	1.50	1.60	±	2.39	1.23	±	1.64
TIA1	1	±	0.15	0.99	±	0.11	0.98	±	0.13
TNFaR1a	1	±	0.11	1.12	±	0.17	0.96	±	0.16
TTR	1	±	0.73[a]	1.75	±	0.42[b]	0.72	±	0.77[a]
UCP2	1	±	0.20[ab]	1.17	±	0.10[a]	0.87	±	0.14[b]

Data are expressed as mean RQ ± SD. Different letters signify the means are significantly different ($p<0.05$) measured by One-way ANOVA followed by Tukey's test for multiple comparisons or Kruskal-Wallis test followed by Dunn's multiple comparison test (if variances were significantly different).
